# Transient and lineage-restricted requirement of Ebf3 for sternum ossification

**DOI:** 10.1242/dev.186239

**Published:** 2020-05-12

**Authors:** Mao Kuriki, Fuminori Sato, Hiroyuki N. Arai, Maina Sogabe, Mari Kaneko, Hiroshi Kiyonari, Koichi Kawakami, Yuki Yoshimoto, Chisa Shukunami, Atsuko Sehara-Fujisawa

**Affiliations:** 1 Department of Regeneration Science and Engineering, Institute for Frontier Life and Medical Sciences, Kyoto University, Kyoto 606-8507, Japan; 2 Laboratory for Animal Resources and Genetic Engineering, RIKEN Center for Biosystems Dynamics Research, Kobe, Hyogo, 650-0047, Japan; 3 Laboratory of Molecular and Developmental Biology, National Institute of Genetics, Department of Genetics, SOKENDAI (The Graduate University for Advanced Studies), Mishima, 411-8540, Japan; 4 Department of Molecular Biology and Biochemistry, Graduate School of Biomedical and Health Sciences, Hiroshima University, Hiroshima, 734-8553, Japan; 5 Department of Muscle Aging and Regenerative Medicine, Research Team for Geriatric Medicine, Tokyo Metropolitan Institute of Gerontology, 35-2 Sakae-cho, Itabashi, Tokyo 173-0015, Japan

**Keywords:** Pre-osteoblast, Sternum, Fibroblast, Runx2, Scleraxis, Lateral plate mesenchyme cells

## Abstract

Osteoblasts arise from bone-surrounding connective tissue containing tenocytes and fibroblasts. Lineages of these cell populations and mechanisms of their differentiation are not well understood. Screening enhancer-trap lines of zebrafish allowed us to identify Ebf3 as a transcription factor marking tenocytes and connective tissue cells in skeletal muscle of embryos. Knockout of Ebf3 in mice had no effect on chondrogenesis but led to sternum ossification defects as a result of defective generation of Runx2^+^ pre-osteoblasts. Conditional and temporal Ebf3 knockout mice revealed requirements of Ebf3 in the lateral plate mesenchyme cells (LPMs), especially in tendon/muscle connective tissue cells, and a stage-specific Ebf3 requirement at embryonic day 9.5-10.5. Upregulated expression of connective tissue markers, such as Egr1/2 and Osr1, increased number of Islet1^+^ mesenchyme cells, and downregulation of gene expression of the Runx2 regulator Shox2 in Ebf3-deleted thoracic LPMs suggest crucial roles of Ebf3 in the onset of lateral plate mesoderm differentiation towards osteoblasts forming sternum tissues.

## Abstract (precis)

**Highlighted Article:** Analysis of conditional and temporal knockout mice reveals roles of Ebf3 in the differentiation of lateral plate mesenchymal cells towards pre-osteoblasts during sternum ossification at the boundary of the lateral and somitic mesoderm.

## INTRODUCTION

The sternum protects the heart and lungs in vertebrates and constitutes the most ventral part of the thoracic skeleton. The sternum first arose in vertebrates during the colonization of land; whereas avians have well-developed sternal tissues, most fish do not have sternum-like bones. The evolution of the sternum highlights its crucial role in forelimb locomotion as a connecting site for the pectoral-muscle tendons (Chen, 1952; Bickley and Logan, 2014).

In general, evidence suggests that osteoblasts differentiate from mesenchymal stem cells (MSCs) that possess the potential to differentiate into osteoblasts, chondrocytes, tenocytes, and perhaps even muscle connective tissue (MCT) cells, fibroblastic cells and adipocytes (Caplan, 1991). A recent study involving single-cell analyses of embryos from embryonic day (E) 9.5-E13.5 enabled visualization of the developmental trajectories of mesenchymal cells into chondrocytes, osteoblasts and MCT cells, suggesting close proximity of the latter two (Cao et al., 2019). However, the cellular hierarchies of mesenchymal differentiation into osteoblasts and MCT cells have not been delineated very well.

Compared with somitic mesenchymal differentiation into axial bones, the differentiation of mesenchyme into sternum bone is relatively simple. All mesenchymal cells in the sternum, including chondrocytes, osteoblasts and proximal connective tissue cells, originate from the lateral plate mesoderm. The existence of osteo-chondro progenitors in the lateral plate mesoderm has been shown by lineage tracing of reporter gene expression activated by paired related homeobox 1(Prx1; also known as Prrx1)-Cre (Akiyama et al., 2005, 2002; Day et al., 2005).

Runt-related transcription factor 2 (Runx2) is an osteoblast master regulator during development and regeneration (Komori et al., 1997; Schroeder et al., 2005). The essential roles of Runx2 in osteo-lineage cells have been well studied, and its activation is defined as the most important indicator of pre-osteoblasts (Komori, 2002; Takarada et al., 2016). Expression of the *Runx2* gene begins in a portion of lateral plate mesenchymal cells (LPMs) at approximately E10.5, which is preceded by Prx1-Cre expression (Bialek et al., 2004; Logan et al., 2002). Thus, the use of Prx1-Cre to study lateral plate mesoderm morphogenesis provides an ideal experimental system to elucidate the molecular/cellular mechanism of the generation of Runx2^+^ pre-osteoblasts from LPMs.

In contrast to mesenchymal differentiation of skeletal components, the cell lineages of skeletal muscle cells have been defined very well, with the identification of the MyoD family and Pax3/Pax7, which are transcription factors involved in the regulation of myogenesis (Relaix et al., 2004). All the skeletal muscle in the trunk, including pectoral muscles, are derived from somites. However, skeletal muscle tissues are complex because different types of MCT cells intercalate into muscle fibers, and these MCT cell lineages are heterogeneous (Giordani et al., 2019). Recent studies on skeletal myogenesis have focused on MCT cells, which play crucial roles in muscle development and maintenance (Sefton and Kardon, 2019). Skeletal muscle cells should colonize a region near the skeletogenic cell components, including osteogenic and chondrogenic cells together with tenocytes and MCT cells, to build the functional locomotorium during development. Thus, the embryonic tenogenic/MCT cells represent a missing link that could be targeted in a proof-of-concept experiment to integrate mechanisms underlying the incorporation of skeletogenic and myogenic cells into the unified locomotorium.

From this idea, we screened enhancer-trap lines of zebrafish and identified the early B-cell factor 3 (Ebf3) as a transcription factor marking scleraxis (Scx)-expressing tenocytes and MCT cells of both zebrafish and mouse embryos. Ebf3 is required for sternum ossification and is required transiently in the Scx^+^ cell lineage of LPMs during the early embryonic stage. Analyses of embryonic LPMs at that stage suggest roles of Ebf3 in the differentiation of osteo-fibrogenic progenitors towards Runx2^+^ pre-osteoblasts at the boundary of lateral and somitic mesoderms.

## RESULTS

### Ebf3 is expressed in tendon/muscle connective tissue progenitors of zebrafish embryos

**Fig. 1.**
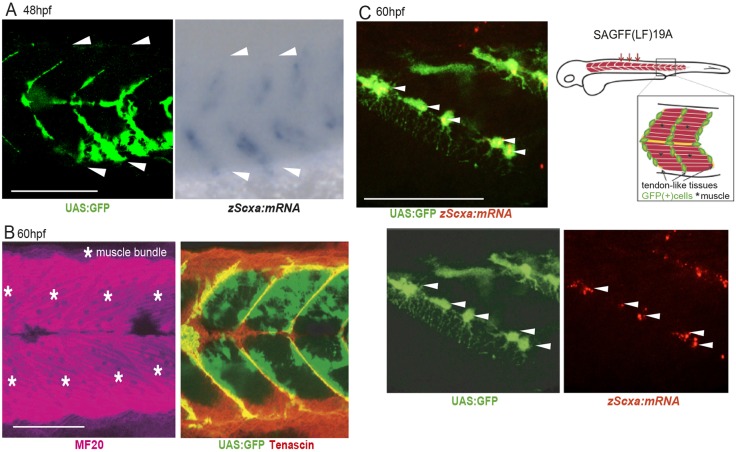
**Identification of an enhancer trap line marking progenitors of tenocytes and interstitial cells in zebrafish embryos.** (A) Expression of UAS:GFP in Gal4-UAS enhancer trap zebrafish line SAGFF(LF)19A and signals for *scxa* mRNA transcripts detected by whole-mount *in situ* hybridization (WISH) at 48 hpf. White arrowheads show segmental boundaries of somites. (B) Whole-mount immunostaining of 60 hpf SAGFF(LF)19A embryos for myogenic (magenta, MF20) and tenogenic (red, tenascin) markers together with UAS:GFP signals at 60 hpf. Asterisks mark muscle bundles. (C) Tenocyte progenitors marked with UAS:GFP and fluorescence *in situ* hybridization for *scxa* (red) at 60 hpf. Note that these cells extended long filopodia through the boundaries of skeletal muscle fibers. Arrowheads indicate UAS:GFP^+^ *zScxa:mRNA*^+^ cells. Schematic shows UAS:GFP^+^ cells in tendon and connective tissues in trunk muscles of SAGFF(LF) 19A line. Scale bars: 100 μm (A,B); 50 μm (C).

We screened Gal4-UAS enhancer trap lines of zebrafish generated previously (Kawakami et al., 2004), in which UAS:GFP expression was found in mesenchymal cells surrounding or adherent to skeletal muscle fibers. One line, SAGFF(LF)19A, showed activated UAS:GFP in a group of cells aligned at the segmental boundary of somites at approximately 48 h post-fertilization (hpf) (Fig. S1A,B, Fig. 1A). In this line, gal4 of SAGFF(LF)19A is trapped upstream of the gene encoding Ebf3. The UAS:GFP signals of SAGFF(LF)19A were detected in Ebf3-positive cells by immunofluorescence staining with an antibody against Ebf3 (Fig. S1C). Furthermore, UAS:GFP^+^ cells were observed in Tenascin^+^ segmental boundaries between myosin heavy chain (MF20)^+^ muscle bundles (Fig. 1B) and developed extremely long filopodia that grew through the boundary of muscle fibers (Fig. 1C). Moreover, *in situ* hybridization revealed that some of these cells co-expressed mRNA for Scleraxisa (Scxa), a tendon-specific transcription factor, showing that UAS:GFP^+^ cells are tendon progenitors (Fig. 1C). MCT cells in muscle connective tissue in close proximity to skeletal muscles were also found to be UAS:GFP^+^ (Fig. 1B). Ebf3^+^ mesenchyme is composed of progenitors of tenocytes and MCT cells in zebrafish embryos.

### Ebf3 is predominantly expressed in the thoracic lateral plate mesenchymal tissues

**Fig. 2.**
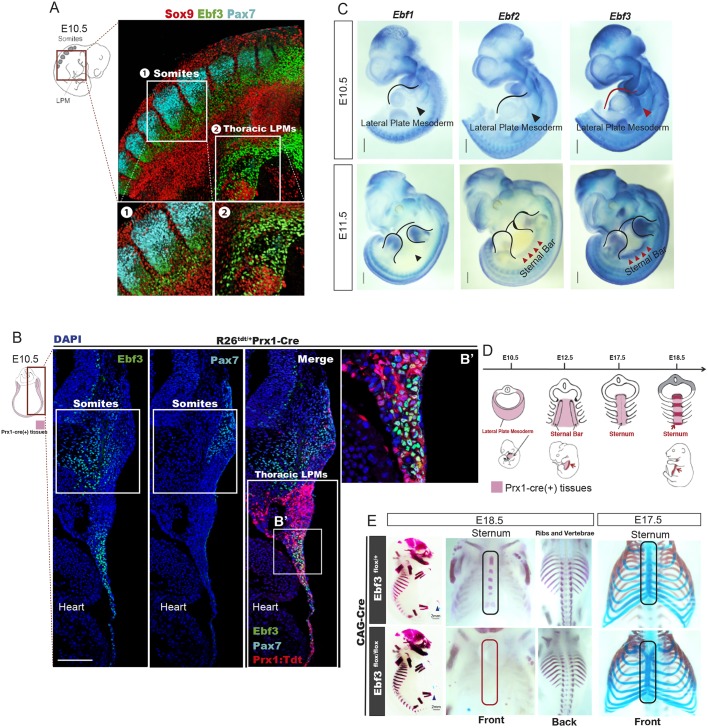
**Essential roles of Ebf3 in sternum ossification.** (A) Whole-mount immunostaining of E10.5 mouse embryo with antibodies against Ebf3 (green), Pax7 (cyan) and Sox9 (red). Ebf3 was expressed in the lateral plate mesoderm, as well as in syndetomes located between Pax7^+^ dermomyotomes and Sox9^+^ sclerotomes. Insets 1 and 2 show higher magnifications of the indicated regions. (B,B′) Ebf3 (green) was expressed in Prx1:Tdtomato^+^ cells (red) at the thoracic lateral plate mesoderm and in syndetomes adjacent to Pax7^+^ dermomyotomes (cyan) at E10.5. B′ shows an enlarged view of the boxed region. (C) WISH for *Ebf1*, *Ebf2* and *Ebf3* in E10.5 and E11.5 mouse embryos. Red arrowheads indicate that *Ebf3*, but not other Ebfs, was expressed in the thoracic lateral plate mesoderm tissue at E10.5 and show highest expression of Ebf3 in the sternal primordial bars at E11.5. (D) Schema of sternum development during murine embryogenesis. Red regions represent sternal bars at E12.5 and ossified zones of the sternum at E18.5. (E) Ossification and chondrogenesis analyses of Ebf3^flox/flox^ CAG-Cre and Ebf3^flox/+^ CAG-Cre embryos at E18.5 and E17.5 based on skeletal preparations. Ebf3-KO mice exhibited defective sternum ossification without any defects in chondrogenesis. Scale bars: 100 μm (B); 1 mm (C); 2 mm (E).

MCT cells play crucial roles in skeletal development. Scx is one of the best-studied key factors that function in tendon development (Cserjesi et al., 1995; Schweitzer et al., 2001; Murchison et al., 2007; Yoshimoto et al., 2017), yet our understanding of MCT and tendon development is still limited. We therefore investigated whether Ebf3 is also expressed in the non-myogenic mesenchyme of mice. Whole-mount immunofluorescence staining of mouse embryos showed that Ebf3 was expressed in the syndetomes, the regions between Pax7^+^ dermomyotomes and Sox9^+^ sclerotomes, and in the thoracic lateral plate mesoderm (Fig. 2A). We crossed Prx1-Cre mice with R26R^Tdtomato/Tdtomato^ mice, as the crossed line marks LPMs in embryos. Analyses of immunofluorescence staining on transverse sections indicated that Ebf3 is expressed in Prx1-Cre R26R^Tdtomato^-activated thoracic LPMs, in addition to the syndetome adjacent to Pax7^+^ dermomyotome (Fig. 2B). Moreover, whole-mount *in situ* hybridization (WISH) revealed the highest expression of *Ebf3* in the thoracic lateral plate mesoderm and sternal primordial bars at E10.5 and E11.5 (Fig. 2C). In contrast, other Ebf transcription factors, *Ebf1* and *Ebf2*, were transcribed in the axial mesenchyme and limb buds but not in the thoracic lateral plate mesoderm at E10.5 (Fig. 2C). *Ebf2* starts to be expressed in sternal primordial bars at E11.5 (Fig. 2C). *Ebf4* was not expressed in mouse embryos at E10.5 and E11.5 (data not shown).

### The regulation of Ebf3 for sternal osteoblast differentiation in the lateral plate mesoderm

**Fig. 3.**
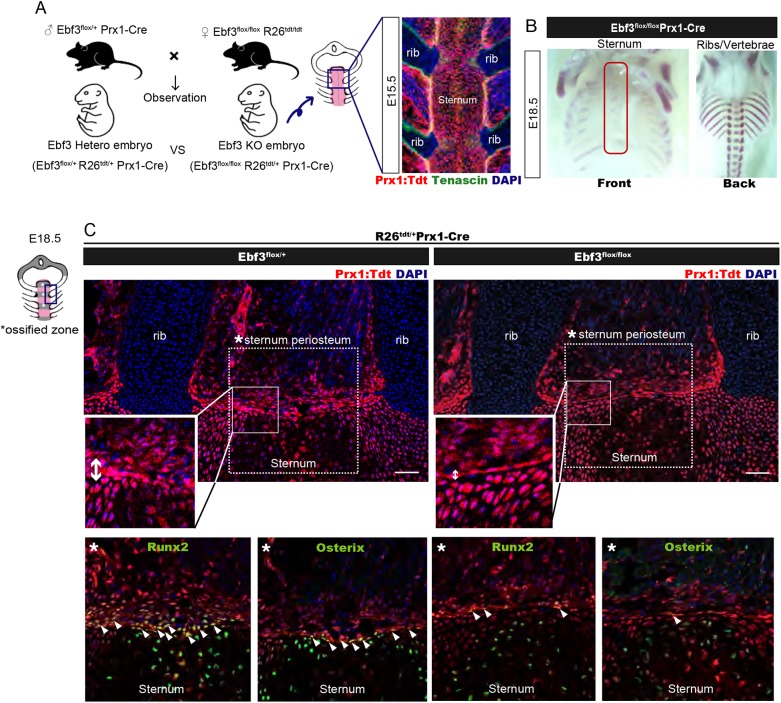
**Ebf3 is required in lateral plate mesoderm-derived cells for sternum osteoblast differentiation.** (A) Left: The generation of lateral plate mesoderm-specific Ebf3-KO mice embryos by crossing the Ebf3^flox/+^ Prx1-Cre line with Ebf3^flox/flox^ Rosa26^Tdtomato/Tdtomato^ mice. Right: The expression pattern of Prx1-Cre-activated Tdtomato in the sternum tissue and muscle connective tissues based on longitudinal sections of E15.5 embryos together with immunostaining for tenascin (green). (B) Ossification analysis based on Alizarin Red skeletal preparations of thoracic bones in Ebf3^flox/flox^ Prx1-Cre embryos at E18.5. (C) Longitudinal sections of the sternum of Ebf3^flox/flox^ Prx1-Cre and Ebf3^flox/+^ Prx1-Cre embryos at E18.5, immunostained for osteoblast (green, osterix) and pre-osteoblast (green, Runx2) markers. Upper panels show the distribution of lateral plate mesoderm-derived cells labeled with Prx1-Cre-activated TdTomato (red) in interstitial cells surrounding the sternum, which was decreased in Ebf3-KO embryos (*n*=7) compared with heterozygous mice (*n*=7). Lower-enlarged panels show much fewer osterix/Runx2-positive lateral plate mesoderm-derived cells in the periosteum of Ebf3-KO mice compared with heterozygous mice (white arrowheads). Scale bars: 100 μm.

To investigate the roles of Ebf3 in development, we prepared conditional Ebf3 knockout (KO) mice (Ebf3^flox/flox^). The targeting vector harbored loxP sites and an FRT-flanked neomycin resistance cassette, in which exon 5 and exon 6 were flanked by a loxP site. These exons encode the DNA-binding domain of Ebf3 (Fig. S2). We generated whole-body Ebf3-KO mice by crossing Ebf3^flox/flox^ mice with Ebf3^flox/+^ CAG-Cre mice. Alizarin Red and Alcian Blue staining revealed that Ebf3-KO embryos had defects in sternal bone formation, but sternal chondrogenesis occurs normally in these mice, with the exception of deformations in the first segment of the sternum (5/5 embryos) (Fig. 2E). Sternal-ossification and chondrogenesis defects were not observed in CAG-Cre Ebf3 heterozygous mice (4/4 embryos). Ossification defects in the third and fourth metatarsal bones were also observed (Fig. S3), whereas ossification of the ribs and vertebrae bones was normal (Fig. 2E). The sternum is derived from thoracic LPMs, whereas ribs and vertebral bones are derived from somitic mesenchymal cells (Durland et al., 2008) (Figs 2D and 3A). To evaluate whether Ebf3 is required for sternum ossification in osteo-fibro lineage cells, we used Prx1-Cre mice, in which osteo-fibro progenitors originating from the lateral plate mesoderm are marked. We crossed Ebf3^flox/flox^ R26^Tdtomato/Tdtomato^ mice with Ebf3^flox/+^ Prx1-Cre mice and generated lateral plate mesoderm-specific conditional Ebf3-KO mice (Fig. 3A). Prx1-Cre Ebf3-KO embryos showed defects in sternum ossification (6/6 embryos), similar to those observed in CAG-Cre general Ebf3-KO embryos (Fig. 3B), whereas no defects were observed in Prx1-Cre Ebf3 heterozygous mice (10/10 embryos), indicating that the Ebf3 expressed in LPMs is required for sternum ossification. We also evaluated lateral plate mesoderm-derived osteoblasts and pre-osteoblasts of the sternum in the Prx1-Cre Ebf3-KO embryos (Ebf3^flox/flox^ R26^Tdtomato/+^ Prx1-Cre) by immunofluorescence staining with antibodies against osterix (Osx; also known as Sp7) and Runx2, respectively. In the ossifying sternum tissue, most Prx1-Cre R26R^Tdtomato^-activated sternum periosteum tissues were occupied by Runx2^+^ pre-osteoblasts in the wild-type and Prx1-Cre Ebf3 heterozygous embryos. Osx^+^ osteoblasts were also present in the sternum periosteum of these mice. In contrast, the numbers of Osx^+^ cells and Runx2^+^ cells in the sternum periosteum decreased dramatically in Prx1-Cre Ebf3-KO embryos (Fig. 3C).

A previous study reported a defect in muscle differentiation of the diaphragm in the Ebf3 Pan-KO mouse embryo (Jin et al., 2014). Because Ebf3 is expressed in Prx1-Cre R26R^Tdtomato^-activated connective tissue cells of the diaphragm and intercostal muscles adjacent to the sternum (Figs S4C and S7C), we examined whether the diaphragm and intercostal skeletal tissues are affected in Prx1-Cre Ebf3-KO mice. The diaphragm and intercostal muscles and tendon/connective tissues adjacent to these muscles appeared normal in Prx1-Cre Ebf3-KO embryos, implying that Ebf3 expressed in lateral plate mesoderm-derived MCT cells are not involved in myogenic/tenogenic defects in these tissues (Fig. S7A,B,D).

### Ebf3 regulates the generation of Runx2^+^ pre-osteoblasts in thoracic LPMs before the onset of the sternal ossification

**Fig. 4.**
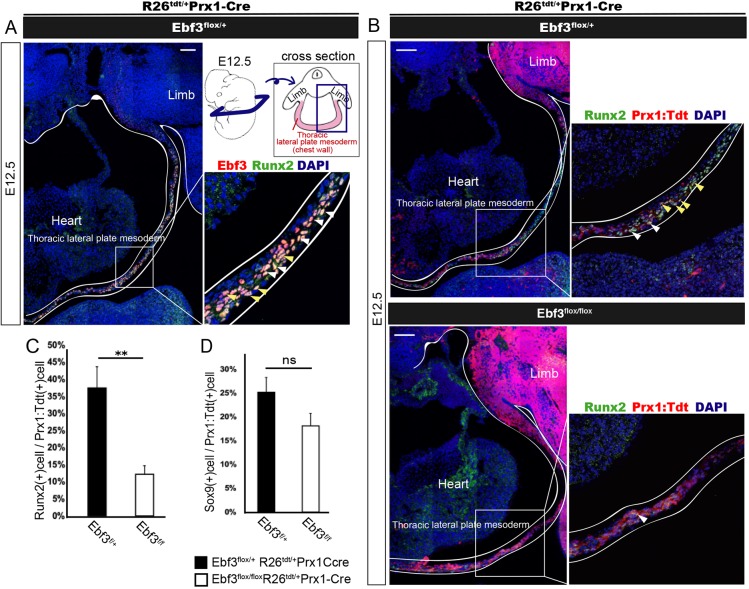
**Decreased number of Runx2^+^ pre-osteoblasts in Ebf3-deficient thoracic lateral plate mesoderm at E12.5.** (A) Transverse section of thoracic lateral plate mesoderm (as indicated in the schematic) at E12.5 immunostained with anti-Ebf3 and anti-Runx2 antibodies. Most but not all Ebf3-expressing cells were Runx2 positive. White and yellow arrowheads show Ebf3/Runx2 double-positive cells. (B) Decrease in the number of Prx1: Tdtomato^+^ Runx2^+^ cells in Ebf3^flox/flox^ Prx1-Cre compared with that in heterozygous embryos (white arrowheads). Immunostained transverse sections of the thoracic lateral plate mesoderm at E12.5 are shown. A and upper panels of B show images of Ebf3/Runx2-double-positive cells and those of Prx1: Tdtomato^+^Runx2^+^ LPMs, respectively, prepared from a single section of a heterozygous embryo stained with antibodies against Ebf3, Runx2 and DAPI. Yellow arrowheads indicate Prx1: Tdtomato^+^Ebf3^+^Runx2^+^ LPMs. (C) The ratio of Runx2^+^ cells in Prx1: Tdtomato^+^ thoracic LPMs was lower in Ebf3^flox/flox^ Prx1-Cre KO mice than in Ebf3 heterozygous mice at E12.5. Error bars represent s.e.m. **P<0.01 (*n*=4). (D) Ratio of Sox9^+^ cells in Prx1: Tdtomato^+^ thoracic LPMs at E12.5. Error bars represent s.e.m.; ns, non-significant (*n*=4). Scale bars: 100 μm.

Ebf3 is expressed in the periosteum and Prx1-Cre R26R^Tdtomato^-activated tendon/connective tissue cells surrounding the sternum, and in connective tissue cells interspersed among intercostal myofibers at the ossification stage (E18.5) (Fig. S4A,C). Ebf3 is undetectable in Osx^+^ or Runx2^+^ osteoblast-lineage cells and tenascin^+^ periosteal cells, suggesting that Ebf3 regulates earlier stages of the osteoblast differentiation than E18.5 (Fig. S4B). There was no significant difference in the number of thoracic LPMs marked with Prx1-Cre R26R^Tdtomato^ between Prx1-Cre Ebf3-KO and heterozygous mice at E12.5 (Fig. S5C). Runx2 is known as the master regulator of lineage commitment from mesenchymal stem cells to pre-osteoblasts (Komori et al., 1997; Schroeder et al., 2005). We found that a large population of thoracic LPMs were Ebf3/Runx2 double positive (Fig. 4A). To determine the roles of Ebf3 in pre-osteoblast differentiation, we investigated expression of Runx2 in the Prx1-Cre R26R^Tdtomato^-activated chest wall of Prx1-Cre Ebf3-KO mice. Compared with Prx1-Cre Ebf3 heterozygous mice, the number of Runx2^+^ cells was significantly lower in Prx1-Cre Ebf3-KO mice (Fig. 4B,C) whereas the numbers of Sox9^+^ cells, the mesenchymal cell population including progenitors of chondrocytes, were similar between these mice (Fig. S5A,B, Fig. 4D). Thus, Ebf3 regulates the number of Runx2^+^ pre-osteoblasts in the thoracic lateral plate mesoderm.

### Ebf3 expression at E9.5-E10.5 is essential for sternal ossification

**Fig. 5.**
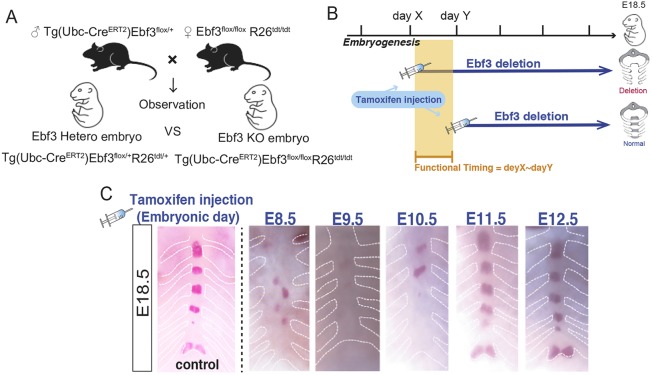
**Ebf3 plays a crucial role in sternum ossification at approximately E9.5-E10.5.** (A) Generation of tamoxifen-induced temporal Ebf3-KO mouse embryos by crossing the Ebf3^flox/+^ Ubc-CreER^T2^ line with Ebf3^flox/flox^ mice. (B) Strategy to determine Ebf3 functional timing in sternal ossification in Ubc-CreER^T2^ Ebf3-KO embryos. (C) Evaluation of sternum phenotypes in Ebf3^flox/flox^ Ubc-CreER^T2^ embryos upon tamoxifen injection at different developmental stages (E8.5-E12.5) based on Alizarin Red skeletal preparations. Embryonic days when tamoxifen was injected into pregnant dams are indicated.

To define the functional timing of Ebf3 expression required for sternum ossification, we generated tamoxifen-induced temporal Ebf3-KO mice by crossing Ebf3^flox/flox^ mice with Ebf3^flox/+^ Ubc-CreER^T2^ mice (Fig. 5A). We then injected tamoxifen into these pregnant dams at different developmental stages (E8.5, E9.5, E10.5, E11.5, E12.5) and evaluated sternal ossification (Fig. 5B). Sternum phenotypes were reproducible only when tamoxifen was injected into these mice at E9.5 (5/5 embryos) or E10.5 (4/5 embryos), but not at E8.5 (6/7 embryos), E11.5 (3/3 embryos), E12.5 (7/7 embryos) or in control (Ubc-CreER^T2^ Ebf3 heterozygous and wild-type) (20/20 embryos and 14/14 embryos) mice, suggesting that Ebf3 functions in sternum ossification mainly at E9.5-E10.5 (Fig. 5C).

### Ebf3 regulates the differentiation of osteo-fibrogenic progenitors

**Fig. 6.**
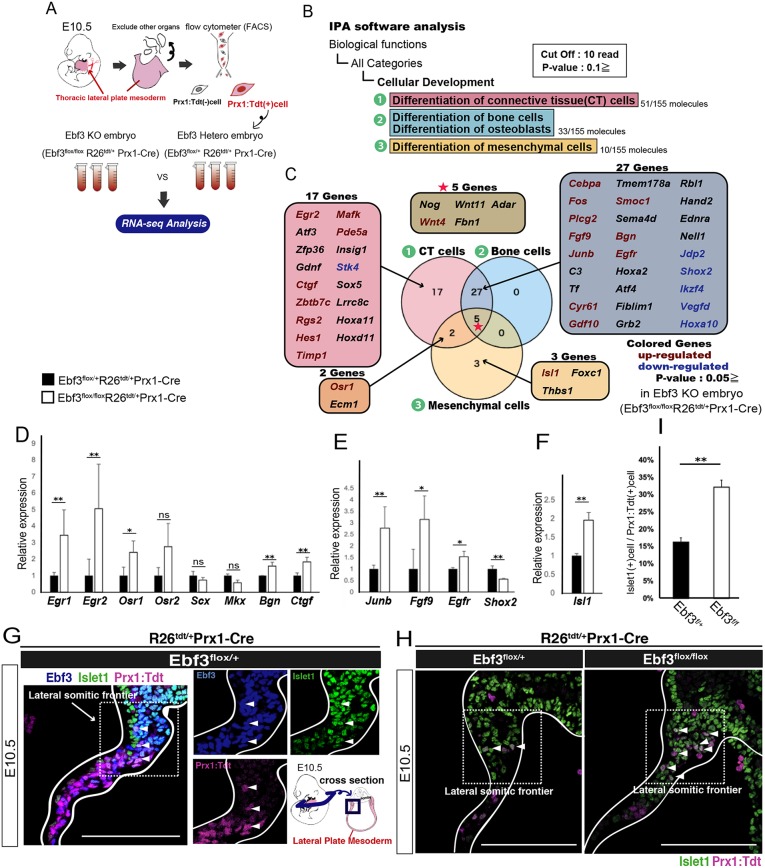
**Ebf3 downstream analysis in mesenchymes of the thoracic lateral plate mesoderm by RNA-seq.** (A) Thoracic LPMs were excised from Ebf3^flox/flox^ Prx1-Cre and Ebf3^flox/+^ Prx1-Cre embryos at E10.5, and Prx1: Tdtomato^+^ cells were isolated by FACS (*n*=3). (B) Differentially expressed genes based on RNA-seq data between Ebf3^flox/flox^ Prx1-Cre and Ebf3^flox/+^ Prx1-Cre embryos (cut off: 10 read, *P*≤0.1) were categorized by their biological functions using IPA software into three groups: (1) genes required for the differentiation of connective-tissue (CT) cells, (2) genes required for the differentiation of bone cells and osteoblasts, and (3) genes required for the differentiation of mesenchymal cells. (C) Venn diagram of these three groups. Genes in red were significantly upregulated and genes in blue were significantly downregulated in Ebf3^flox/flox^ Prx1-Cre compared with expression in Ebf3^flox/+^ Prx1-Cre embryos (*P*<0.05) (from RNA-seq data). (D) Relative expression levels of MCT cell/tenocyte-associated genes in Ebf3^flox/flox^ Prx1-Cre embryos compared with those in Ebf3^flox/+^ Prx1-Cre embryos (from RNA-seq data). **P<0.01, *P<0.05; ns, not significant (*n*=3). (E) Relative expression levels of osteoblast-associated genes in Ebf3^flox/flox^ Prx1-Cre embryos compared with those in Ebf3^flox/+^ Prx1-Cre embryos. **P<0.01, *P<0.05 (from RNA-seq data) (*n*=3). (F) Relative expression level of *Islet1* in Ebf3^flox/flox^ Prx1-Cre embryos was increased significantly compared with that in Ebf3^flox/+^ Prx1-Cre embryos. **P<0.01 (from RNA-seq data) (*n*=3). (G) Islet1 was expressed in Prx1:Tdtomato^+^ Ebf3^+^ cells at the lateral somitic frontier of the thoracic lateral plate mesoderm (as indicated in the schematic) at E10.5 (white arrowheads). (H) Increase in the number of Prx1:Tdtomato^+^ Islet1^+^ cells in Ebf3^flox/flox^ Prx1-Cre (*n*=3) compared with that in heterozygous embryos (*n*=3) (white arrowheads). Immunostained transverse sections of the lateral somitic frontier at E10.5 are shown. (I) The ratio of Islet1^+^ cells in Prx1:Tdtomato^+^ LPMs was higher in Ebf3^flox/flox^ Prx1-Cre compared with that in heterozygous mice at E10.5. **P<0.01 (*n*=3). Error bars represent s.e.m. Scale bars: 100 μm.

To elucidate how Ebf3 regulates the differentiation of thoracic LPMs at E10.5, we compared transcripts in thoracic LPMs isolated from Prx1-Cre Ebf3-KO mice with those from Prx1-Cre Ebf3 heterozygous mice. Trunks without limb buds, heart and other organs in the thoracic/abdominal cavity were isolated, and then thoracic wall tissues were excised. Prx1-Cre R26R^Tdtomato^-activated LPMs were isolated from these thoracic wall tissues by fluorescence-activated cell sorting (FACS), and RNA-seq analysis was performed (Fig. 6A).

Downstream analysis was performed for this RNA-seq data using Ingenuity Pathway Analysis (IPA) software. In the IPA Knowledge Base derived from omics data, annotated genes were categorized by their biological functions. In the category of ‘Cellular Development’, we focused on three groups associated with the differentiation of (1) connective tissue (CT) cells, (2) bone cells/osteoblasts and (3) mesenchymal cells (Fig. 6B). The Venn diagram in Fig. 6C shows the differentially expressed genes annotated in these three groups, in which genes in red and blue are those significantly up- and downregulated, respectively, in Prx1-Cre Ebf3-KO mice (Fig. 6C). Most of the differentially expressed genes were found in (1) CT cells and the merged region of (1) CT cells and (2) bone cells (Fig. 6C).

These data provide insight into genes associated with the sternum ossification phenotype. Increased transcript levels of genes that regulate the formation of tendon and MCTs, such as early growth response protein 1 (*Egr1*), early growth response protein 2 (*Egr2*) and odd-skipped related gene 1 (*Osr1*), were found in Prx1-Cre Ebf3-KO embryos (Fig. 6D). The genes encoding biglycan (Bgn), a proteoglycan involved in maintaining tendon-progenitor pools, and connective tissue growth factor (Ctgf; also known as Ccn2) were also upregulated in Prx1-Cre Ebf3-KO embryos (Fig. 6D). We also confirmed an increase in expression of the Jun B proto-oncogene (*Junb*), fibroblast growth factor 9 (*Fgf9*) and epidermal growth factor receptor (*Egfr*), which regulate proliferation and maintain undifferentiated states during the initial steps of osteoblast differentiation (Fig. 6E). However, transcripts of the short stature homeobox 2 (*Shox2*) gene, encoding a site-specific regulator of Runx2 during long-bone formation, were decreased in Prx1-Cre Ebf3-KO mice (Fig. 6E).

In contrast, genes encoding tendon-associated transcription factors such as Scx and its upstream factor homeobox protein mohawk (Mkx) were expressed similarly in Prx1-Cre Ebf3-KO and control embryos (Fig. 6D). The expression levels of collagen genes, such as those encoding collagen types I, III and VI did not differ between Prx1-Cre Ebf3-KO and heterozygous embryos (Fig. S6A). The expression of regulatory genes involved in chondrocyte differentiation, such as the Sry-related HMG box (Sox)-family genes (*Sox9*, *Sox5*, *Sox6*) were unchanged in Prx1-Cre Ebf3-KO embryos (Fig. S6B). These results suggest that progenitors of pre-osteoblasts remain undifferentiated and express MCT markers in the absence of Ebf3.

### Ebf3 regulates the differentiation of Scx-Cre lineage cells

**Fig. 7.**
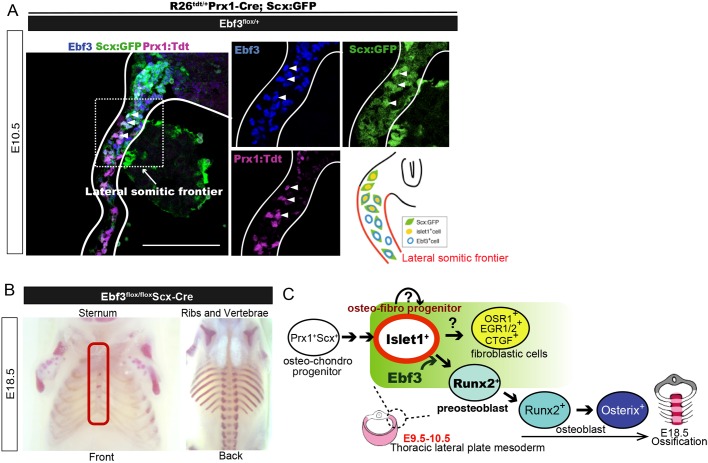
**Ebf3 regulates mesenchymal progenitors in Scx-Cre lineage cells of the thoracic lateral plate mesoderm.** (A) Ebf3 was expressed in Prx1:Tdtomato^+^ Scx:GFP^+^ cells at the lateral somitic frontier of the thoracic lateral plate mesoderm at E10.5 (white arrowheads). Scale bar: 100 μm. Schematic shows Ebf3, Scx:GFP and Islet1 expression in the lateral somitic frontier. (B) Ossification analysis based on Alizarin Red skeletal preparations of thoracic bones in Ebf3^flox/flox^ Scx-Cre embryos at E18.5. Red rectangle highlights ossification defects of the sternum. (C) Schematic summarizing the working hypothesis on the roles of Ebf3 in sternum ossification. Osteo-chondrogenic progenitors in the lateral plate mesoderm give rise to osteo-fibro progenitors marked with Islet1^+^ cells in the lateral somitic frontier (green area). Ebf3 regulates differentiation of these cells towards Runx2^+^ pre-osteoblasts (light blue), which differentiate into osteoblasts and osteocytes to form the sternum bone. In the absence of Ebf3, osteo-fibro progenitors differentiate towards fibroblastic cells (yellow) or self-renew.

Previous studies proposed the term ‘primaxial’ to define the domain of the uniform somitic cell population and ‘abaxial’ to define the domain where somite-derived cells differentiate in the context of lateral plate-derived MCT. The boundary region between these two domains is termed ‘the lateral somitic frontier’ (Durland et al., 2008; Burke and Nowicki, 2003). One upregulated gene, insulin gene enhancer protein 1 (*Islet1*; *Isl1*) (Fig. 6F), encodes a LIM-homeodomain protein and was expressed in the lateral somitic frontier; furthermore, its expression overlapped with that of Ebf3 (Fig. 6G). Moreover, the number of Islet1^+^/Prx1-Cre:TdTomato^+^ cells was significantly higher in Prx1-Cre Ebf3-KO than in Prx1-Cre Ebf3 heterozygous embryos (Fig. 6H,I). In contrast, no change in Scx expression was observed in Prx1-Cre Ebf3-KO mice (data not shown). During mouse embryogenesis, Scx transcripts are first detected between E9.5 and E10.5 in the sclerotome, mesenchymes of the body wall, and limb buds. As development proceeds, the expression of Scx is gradually restricted to regions where tendon and ligament tissues are formed (Cserjesi et al., 1995). When Scx:GFP R26R^Tdtomato/Tdtomato^ mice were crossed with Ebf3^flox/+^ Prx1-Cre mice, Scx:GFP was co-expressed in Ebf3^+^ cells in some TdTomato^+^ LPMs (Fig. 7A). To determine whether Ebf3 is required in Scx^+^ LPMs, Ebf3^flox/flox^ mice were crossed with Ebf3^flox/+^ Scx-Cre mice. We found defective sternum ossification in Ebf3^flox/flox^ Scx-Cre mice (6/6 embryos) but not in the heterozygous mice (7/7 embryos). This suggests that sternum ossification requires Ebf3 in Scx-Cre lineage cells located at the lateral somitic frontier in E9.5-E10.5 mice (Fig. 7B).

## DISCUSSION

In this study, we demonstrate that Ebf3 plays a pivotal role in sternum ossification. Runx1 and Runx2 cooperatively regulate sternal morphogenesis and the commitment of LPMs to differentiate into sternal chondrocytes specifically; sternum tissue has been found to be completely absent in Prx1-Cre-induced Runx1/Runx2 double-KO mice (Kimura et al., 2010). In contrast, CAG-Cre-, Prx1-Cre- or Scx-Cre-driven Ebf3 deletion resulted in defective ossification of the sternum without affecting its chondrogenesis. Thus, specific roles in sternum ossification, but not in chondrogenesis, are a unique feature of Ebf3 compared with many other genes that regulate sternum formation.

The effects of Ubc-CreER^T2^-driven Ebf3 deletion were extremely informative because they clearly indicated that the timing of Ebf3 function in sternum ossification is E9.5-E10.5. E9.5 approximately corresponds to the earliest stage of limb bud formation, whereas E10.5 is the stage when Runx2 starts to be expressed in a proportion of the LPMs along the rib cage. This raises the question of what types of mesenchymal cell differentiation are regulated by Ebf3 to produce pre-osteoblasts. A comparison of RNA-seq results between thoracic LPMs isolated from Prx1-Cre-driven Ebf3-KO mice and those from control embryos at E10.5 demonstrated increases in MCT cell/tenocyte-associated transcripts, such as *Egr1*, *Egr2* and *Osr1* (Lejard et al., 2011; Vallecillo-García et al., 2017), as well as a set of proliferating mesenchyme markers, including *Islet1*, *Junb*, *Fgf9* and *Egfr* in the Ebf3-KO LPMs (Kenner et al., 2004; Yang et al., 2006; Zhu et al., 2011; Ornitz and Marie, 2015; Linder et al., 2018). In contrast, the expression of *Shox2*, which regulates the formation of proximal appendicular bones and is found as a factor upstream of Runx2, was significantly lower in Ebf3-KO than in control LPMs. Moreover, an increased number of Islet1^+^ cells was observed in Ebf3-KO embryos. Thus, mesenchymal differentiation before the activation of Runx2 is affected by the lack of Ebf3, suggesting that Ebf3 is crucial for generating Runx2^+^ pre-osteoblasts from their progenitors in the thoracic lateral plate mesoderm. Fig. 7C shows our working hypothesis on the roles of Ebf3 in sternum ossification. Because sternal chondrogenesis was not affected in Ebf3-KO mice, osteo-chondrogenic progenitors are located upstream of osteo-fibrogenic progenitors in the hierarchy of the lateral plate mesoderm differentiation in this hypothesis.

However, the exact identity of osteo-fibrogenic progenitors awaits further characterization of LPMs. Islet1 expression can mark mesenchymal stem cells in the lateral plate mesoderm rather than osteo-fibrogenic progenitors. Lineage and single-cell analyses of LPMs will elucidate more precise roles for Ebf3 in their differentiation and determine whether Ebf3 promotes the differentiation of LPMs into pre-osteoblasts by inhibiting the self-renewal of Islet1^+^ LPMs or whether these cells acquire alternative fates towards cells other than pre-osteoblasts in the absence of Ebf3. The mechanism of how Ebf3 mediates the differentiation of LPMs towards pre-osteoblasts as a transcription factor also needs to be elucidated. Recent studies on the roles of Ebf1 in B-cell development suggest roles for Ebf1 in epigenetic events, in addition to transcriptional regulation, suggesting that members of the Ebf family can dynamically modulate the chromatin structure (Boller et al., 2016; Li et al., 2018).

Notably, Ebf3^+^ Islet1^+^ cells are found in the lateral somitic frontier. Pax3^+^ muscle progenitors begin to cross this boundary at E9.5, differentiate into MyoD^+^ myoblasts from E10.5 (Relaix et al., 2004), and form abaxial muscles in the lateral plate mesoderm microenvironment, which may differ from that of the somitic mesoderm (Durland et al., 2008; Burke and Nowicki, 2003). The requirement of Ebf3 at E9.5-E10.5, which was determined using Ubc-CreER^T2^ mice, indicates that the differentiation of LPMs towards pre-osteoblasts starts at the lateral somitic frontier.

Recent studies have indicated heterogeneity of fibroblastic cells in skeletal muscle, including some Scx^+^ or Osr1^+^ cells (Yoshimoto et al., 2017; Vallecillo-García et al., 2017). These Scx^+^ cells might include the lateral plate mesoderm-derived cells observed in this study. Further characterization of MCT cells is essential to verify that Ebf3 is required for the differentiation of some types of MCT cells.

Vertebrates developed the sternum during evolution, providing attachment sites for the pectoral muscles that generate the force used for walking or flying (Chen, 1952; Bickley and Logan, 2014). In contrast, the sternum is lacking in most fish, including zebrafish. Nonetheless, Ebf3 is expressed in progenitors of tenocytes and MCT cells in both zebrafish and mice. Given that Scx genes are required for ossification of ventrally elongated precaudal vertebrae in zebrafish (Kague et al., 2019), it would be interesting to investigate whether Ebf3 is required in fish also for abaxial bone formation or other morphogenesis of LPMs.

In this study, we found crucial roles of Ebf3 in sternum ossification. Whereas members of the Ebf family are expressed redundantly in the somitic mesoderm and limb buds, expression of Ebf3 predominates over others in thoracic LPMs, which could explain the defective sternum ossification caused by the lack of Ebf3 in LPMs. In contrast to a previous observation of thickened diaphragm with myogenic disorders in Ebf3 Pan-KO mice (Jin et al., 2014), the myogenesis of diaphragm and intercostal muscle was minimally affected in Prx1-Cre lateral plate mesoderm-specific Ebf3-KO mice, suggesting that Ebf3 expressed in somitic MCTs is required for this myogenesis, or that other members of the Ebf family expressed redundantly in somitic and lateral plate mesoderm can compensate for the lack of Ebf3 in myogenesis.

In summary, Ebf3 expressed in Prx-Cre- and Scx-Cre-activated lineage cells mediates sternum ossification by regulating differentiation of LPMs towards pre-osteoblasts at the lateral somitic frontier (Fig. 7C). The number of Islet1^+^ mesenchymal cells and expression of connective tissue cell/tenocyte-associated genes, such as *Egr1*, *Egr2* and *Osr1*, were increased in Ebf3-deleted thoracic LPMs. How Ebf3 regulates production of Islet1^+^ cells and the fate decision of LPMs into osteoblasts and fibroblasts remain open questions. Identification of target genes for Ebf3, for example by ChIP analyses, together with single cell analyses of LPMs at the early embryonic stages of wild-type and Ebf3-KO mice will be required for a more detailed understanding of the function of Ebf3 in LPM differentiation.

## MATERIALS AND METHODS

### Analysis of zebrafish embryos

Embryos were fixed in 4% paraformaldehyde (PFA)/PBS overnight at 4°C, rinsed in PBS-T (PBS with 0.05% Tween 20), and exposed to 0.2% Triton X-100/PBS. For whole-mount immunostaining, embryos were incubated in Blocking One solution (Nacalai Tesque), washed with 0.2% Triton X-100/PBS, and stained with the following primary antibodies: anti-Ebf3 (1:1000; R&D Systems, AF5166), anti-Tenascin (1:1000; Millipore, AB19013), anti-MF20 [1:200; Developmental Studies Hybridoma Bank (DSHB)]; Alexa Fluor-conjugated antibodies (1:1000; Thermo Fisher Scientific, A-21203, A-21206) were used as secondary antibodies. For WISH, GFP-marked RNA probes were generated from the cDNA of *scxa* amplified by reverse transcription PCR (RT-PCR) with the following PCR primers: Scxa forward primer, 5′-ACCGGCTCCGAGCCGATACGTGTA-3′; Scxa reverse primer, 5′-GGTTGCTGAGGCAGAATGTGCAGA-3′.

### Generation of Ebf3 conditional knockout mice

To generate Ebf3-floxed mice (accession number CDB1070K: http://www2.clst.riken.jp/arg/mutant%20mice%20list.html), a targeting vector harboring loxP sites as well as an FRT-flanked neomycin resistance cassette, in which exons 5 and 6 were flanked by a loxP site, were electroplated into TT2 embryonic stem cells (ESCs) (Yagi et al., 1993). After cell colony isolation by G418 and Southern blot analysis, ESCs containing the floxed allele were injected into 8-cell stage embryos to generate chimeric mice. The FRT-flanked Neo cassette was removed by mating with Flpe mice. Ebf3^flox/flox^ mice were backcrossed at least ten times with mice of the C57BL/6NCrSlc background before analysis (Fig. S2). These mice were crossed with CAG-Cre, Prx1-Cre (Jackson Laboratory), Ubc-CreER^T2^ or Scx-Cre mice to generate CAG-Cre Ebf3^flox/+^ mice, Prx1-Cre Ebf3^flox/+^ mice, Ubc-CreER^T2^ Ebf3^flox/+^ mice, or Scx-Cre Ebf3^flox/+^ mice, and their progeny were inter-crossed with Ebf3^flox/flox^:Rosa26R^TdTomato^ mice to obtain CAG-Cre or Prx1-Cre or Ubc-CreER^T2^, Ebf3^flox/flox^ Rosa26^Tdtomato/+^ mice.

### Other mouse strains

Mouse strains including CAG-Cre, Prx1-Cre, Ubc-CreER^T2^ and Rosa26R:TdTomato were previously described (Logan et al., 2002; Sakai and Miyazaki, 1997; Ruzankina et al., 2007; Madisen et al., 2010). These mice were obtained from the Jackson Laboratory. Scx-Cre mice and Scx:GFP mice (Sugimoto et al., 2013) were previously generated (Sugimoto et al., 2013). All mouse strains were maintained in a C57BL/6NCrSlc background and their genotypes were determined by PCR (Fig. S2). All experiments involving animals were performed according to the guidelines of the animal care committee of Kyoto University and the Institutional Animal Care and Use Committee (IACUC) of RIKEN Kobe Branch, and conformed to relevant guidelines and laws.

### Tamoxifen injection

To induce CreER^T2^-mediated recombination in Ubc-CreER^T2^ Ebf3^flox/flox^ embryos, pregnant dams were treated with 0.1 ml tamoxifen solution (10 mg/ml in peanut oil; Sigma-Aldrich) by gavage on each embryonic day, and embryos were analyzed at E18.5.

### Whole-mount *in situ* hybridization

For WISH, digoxigenin-labeled antisense riboprobes were transcribed from cloned gene-specific probes (DIG RNA Labeling Kit, Roche). Whole embryos were fixed overnight in 4% PFA/PBS. Embryos were fixed, washed in PBT (PBS containing 0.1% Tween 20) and dehydrated stepwise in 25%, 50% ,75% ethanol/PBT and 100% ethanol at 4°C. The embryos were rehydrated by reversing the ethanol/PBT steps and washing with PBT. After treating the embryos with 10 mg/ml Protein K in PBT at 37°C for 20 min, they were incubated in 2 mg/ml glycine in PBT at room temperature (RT), washed in PBT, re-fixed for 20 min with 4% PFA/PBS and 0.2% glutaraldehyde in PBT, washed in PBT, and finally bleached in 6% H_2_O_2_ in PBT. After further washing with PBT, the embryos were incubated in pre-hybridization solution (50% formamide, 5× SSC, 1% SDS, 50 mg/ml yeast tRNA, 50 mg/ml heparin) at 65°C for 2 h. Subsequently, the embryos were incubated in probe solution/pre-hybridization solution (pre-hybridization solution with 1:1000 digoxygenin) overnight at 65°C. Unbound probes were removed through a series of washing steps with Solution 1 (50% formamide, 5× SSC, 1% SDS), Solution 2 (5× SSC, 0.1% Tween 20) and Solution 3 (50% formamide, 2× SSC, 1% SDS) at 70°C for 30 min. After washing with TBST (150 mM NaCl, 100 mM Tris-HCl, 1% Tween 20), embryos were blocked for 90 min at RT in 1.5% Blocking-Reagent/TBST (Roche), followed by incubation at 4°C overnight in 1.5% Blocking-Reagent/TBST with digoxigenin antibody conjugated with alkaline phosphatase (1:3000; 11 093 274 910, Roche). Unbound antibody was removed by washing at RT with TBST, and the samples were stained with BM Purple AP Substrate (Roche).

### Immunostaining

For immunostaining, whole embryos were harvested at E9.5-E18.5 in PBS and fixed in 4% PFA/PBS. Embryos at E9.5-E14.5 were fixed for 1-3 h at RT. Embryos at E15.5-E18.5 were fixed overnight at 4°C. The embryos were then rinsed with PBS and immersed in 5% sucrose/PBS for 30 min, in 15% sucrose/PBS overnight, and in 30% sucrose/PBS overnight at 4°C prior to embedding in OCT media (Leica Microsystems). Embedded tissues were sliced using a Cryostat (Leica CM 3050 S) at a thickness of 10 µm. After washing with PBS-T (PBS with 0.05% Tween 20), the embryo tissues were blocked for 60 min at RT in Blocking One (Nacalai Tesque) and stained with primary antibodies for Ebf3 (1:1000; R&D Systems, AF5166), Sox9 (1:1000; Millipore, AB5535), tenascin (1:1000; Millipore, AB19013), GFP (1:500; Nacalai Tesque, 04404-84), osterix (1:1000; ab22552, Abcam), Runx2 (1:500; Abcam, ab23981), Islet1 (1:1000; Abcam, ab109517), MF20-c (1:200; DSHB, AB_214781) and laminin (1:1000; Sigma-Aldrich, L9393). Followed by washing in PBS-T, Alexa Fluor-conjugated secondary antibodies (1:1000; Thermo Fisher, A-31571, A-21206, A-21447, A-21208) were used. Tissue samples were imaged using a Leica SP8 confocal microscope. The tiling of images of stained sections was performed automatically using LAS X software (Leica). Evaluation of the cell number was carried out with ImageJ software.

### Skeletal preparations

Skeletal preparation of embryos was performed as previously described (McLeod, 1980).

### RNA-seq analysis

To evaluate the developmental function of Ebf3 in the thoracic lateral plate mesoderm comprehensively, we analyzed global gene expression changes by RNA-seq analyses in thoracic LPM samples collected from Prx1-Cre homozygous and heterozygous Ebf3-KO mice at E10.5. Prx1-Cre R26RTdtomato-activated cells were isolated from each tissue by FACS. Total RNA was extracted from the cells isolated by FACS in cell lysis buffer (TRIzol LS Reagent, Life Technologies). RNA samples were analyzed with an Agilent Bioanalyzer RNA 6000 Pico Kit (5067-1513) and subjected to library preparation according to the Takara Bio SMARTer kit and DNA High-sensitivity kit (5067-4626). Sequencing was performed with the Illumina NextSeq-500 platform, which generated raw sequencing reads. Reads were mapped to the reference genome and transcript annotation was performed (Mouse, mm 10, 49,585 genes, 103,982 transcripts) (ENSEMBLE, provided by CLC). Using IPA software (Qiagen), downstream effects analysis was performed for RNA-seq data.

### Statistical analysis

All the data are presented as the mean±s.d. (*n*≥3). Statistical significance was assessed using Student's *t*-test.

## Supplementary Material

Supplementary information

Reviewer comments
