# Supplementary material for: Transient and lineage-restricted requirement of Ebf3 for sternum ossification
Source: Development. 2020 May 12;147(9):dev186239. doi: 10.1242/dev.186239 (PMC7240299; doi:10.1242/dev.186239)
Supplement: Supplementary information [file develop-147-186239-s1.pdf]

A

SAGFF(LF)19A Zebrafish Line

Informations: website (https://kawakami.lab.nig.ac.jp/)
Laboratory of Molecular and Developmental Biology,
National Institute of Genetics, Department of Genetics,
SOKENDAI (The Graduate University for Advanced Studies), Mishima, Japan

B

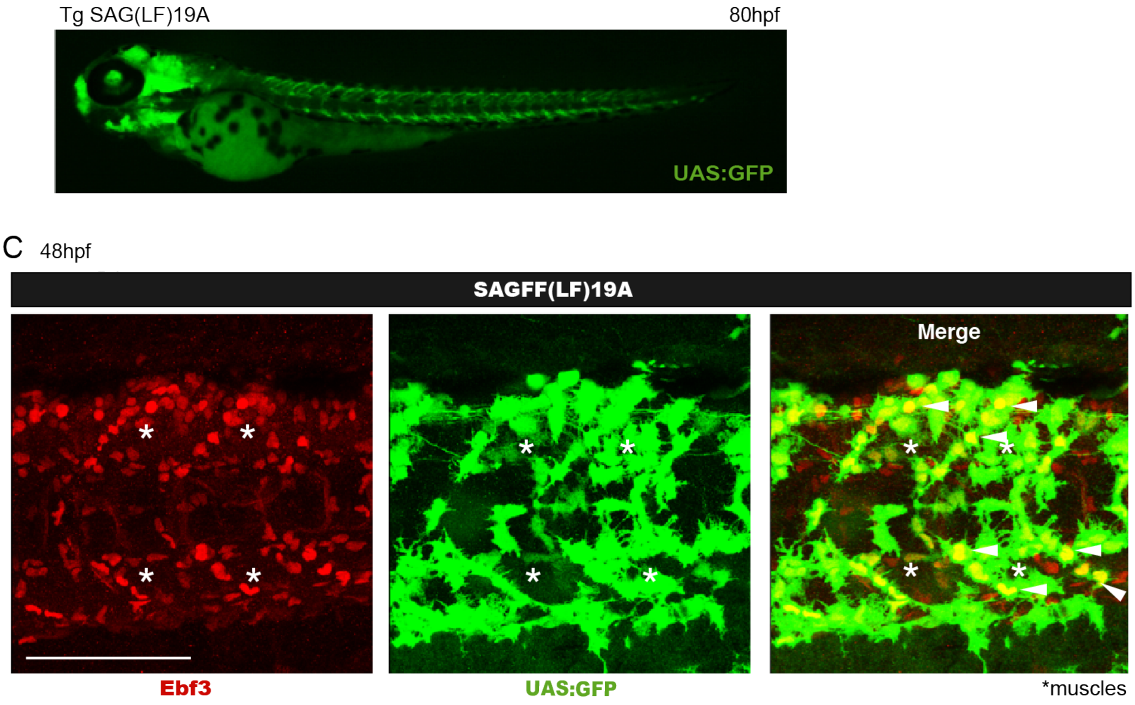

Figure S1.

Zebrafish line SAGFF(LF)19A drives UAS:GFP in Ebf3-positive cells

A) Information about zebrafish line SAGFF(LF)19A.

B) Whole-body expression pattern of UAS: GFP signals in SAGFF(LF)19A at 80 hpf.

C) Whole-mount immunostaining of SAGFF(LF)19A embryos for Ebf3 (red) with UAS:GFP signals at 48 hpf (scale bar, 100μm).

Development • Supplementary information

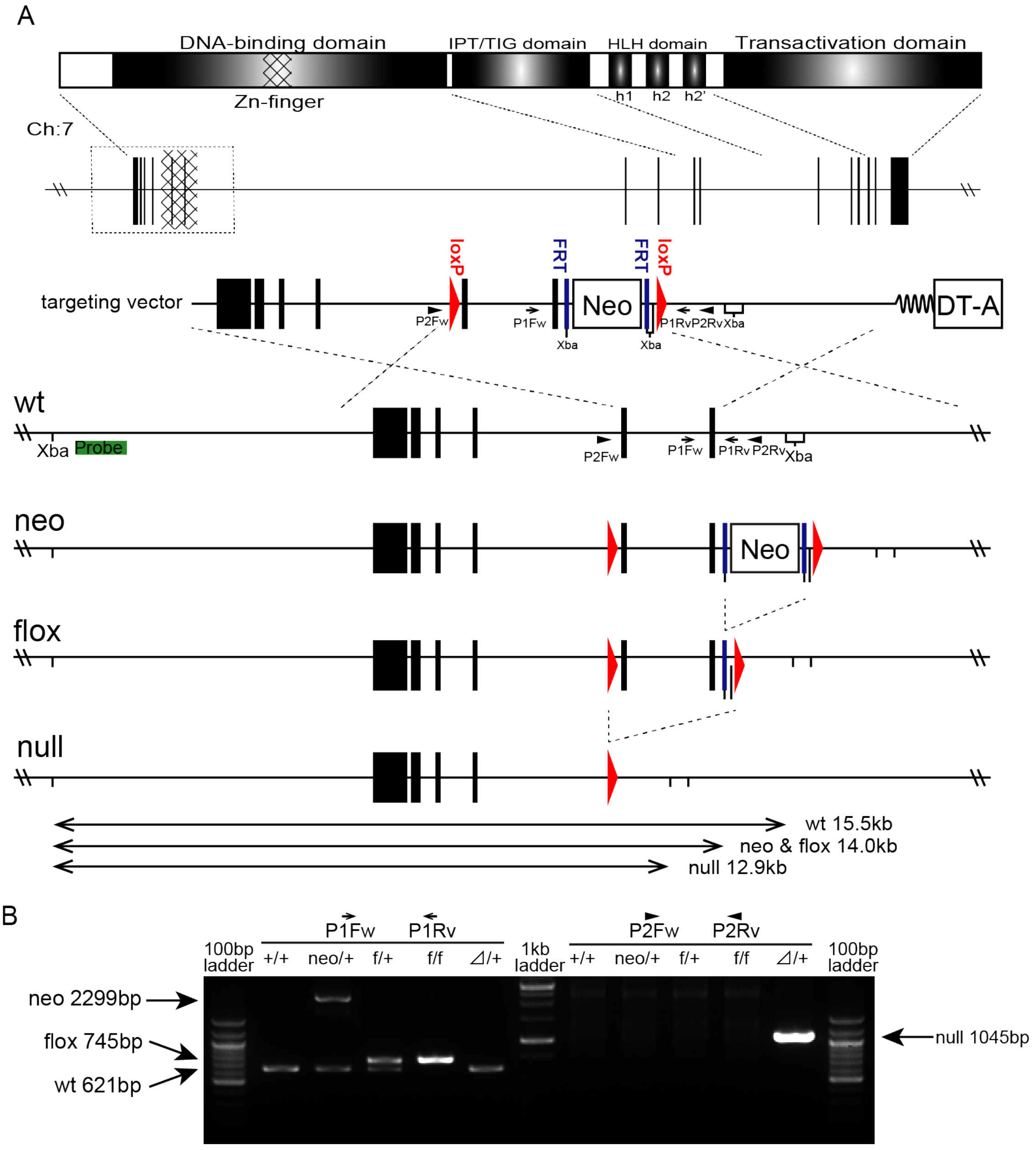

C

Primer	5' Label	Sequence 5' ⟶ 3'	3' Label
P1Fw(Forward)		CTC CTA CTG CTT CAT GGT CAG ACT	
P1Rv(Reverse)		GCA GAT TAA ATT CAT TCT ACC ACT GG	
P2Fw(Forward)		GGC GTG AGC GCA CCT AGG GCT TCG CA	
P2Rv(Reverse)		ATC TGC AGT CAA TCA CAC ACT GGG CC	

Development • Supplementary information

Figure S2.

Generation of Ebf3$^{flox/flox}$ mice and primers used for genotyping

A) Generation of Ebf3$^{flox/flox}$ mice.

B) Genotyping to characterize wild-type mice, Ebf3-floxed mice (Ebf3$^{flox/flox}$, Ebf3$^{flox/+}$), and Cre-activated Ebf3-KO mice (Ebf 3 $^{\Delta/\Delta}$, Ebf 3 $^{\Delta/+}$) by PCR.

C) Primers used for PCR in mouse lines shown in Figure S2B.

Development • Supplementary information

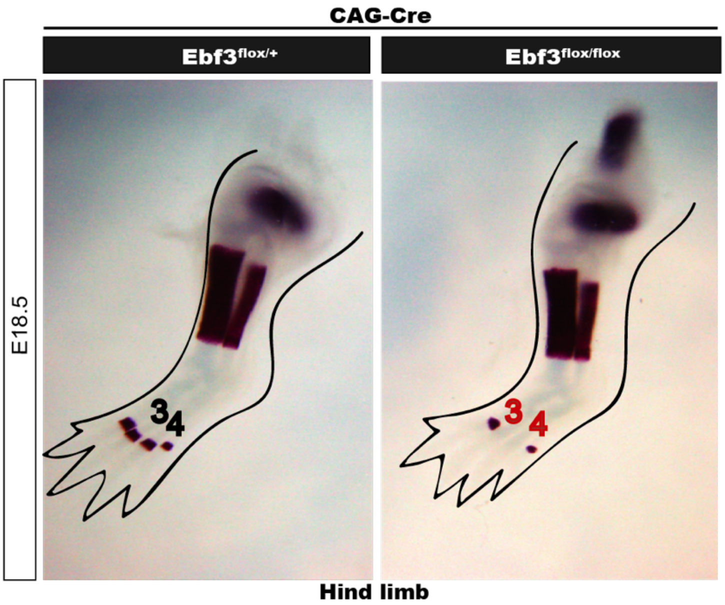

Figure S3.

Essential roles of Ebf3 in ossification of metatarsal-bones

Ossification analysis of hind-leg bones in Ebf3$^{flox/flox}$ CAG-Cre and Ebf3$^{flox/+}$CAG-Cre embryos at E18.5 based on Alizarin-red skeletal preparations. Ossification defects in the third and fourth metatarsal bones were observed in Ebf3$^{flox/flox}$ CAG-Cre.

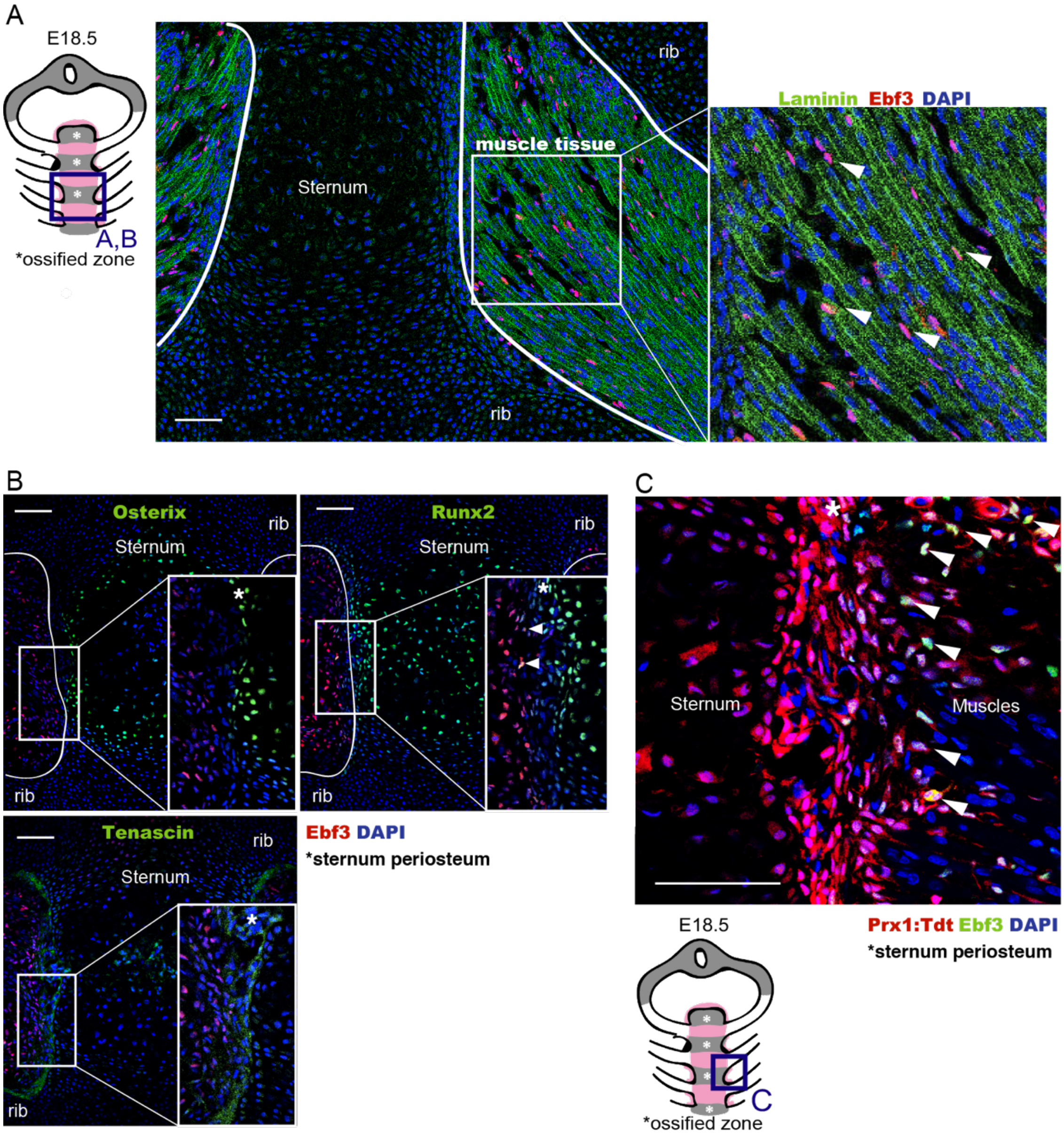

Figure S4.

Ebf3 is expressed in muscle connective tissues, not in osteoblasts in the sternum at ossification stages

A) Immunostaining of longitudinal sections of sternum tissues for Laminin (green) and Ebf3 (red) at E18.5. White arrows show $Ebf3^{+}$ cells in muscle connective tissue (MCT) (scale bar, 100μm).

B) Immunostaining of longitudinal sections of sternum tissues for an osteoblast marker (green, Osterix), pre-osteoblast marker (green, Runx2), and periosteum marker (green, Tenascin) at E18.5 (scale bar, 100μm).

C) Immunostaining for $Ebf3^{+}$ cells in $Prx1{:}Tdtomato^{+}$ MCTs surrounding the sternum at E18.5 based on transverse section of Prx1-Cre $R26^{Tdt/+}$ (white arrows) (scale bar, 100μm).

Development • Supplementary information

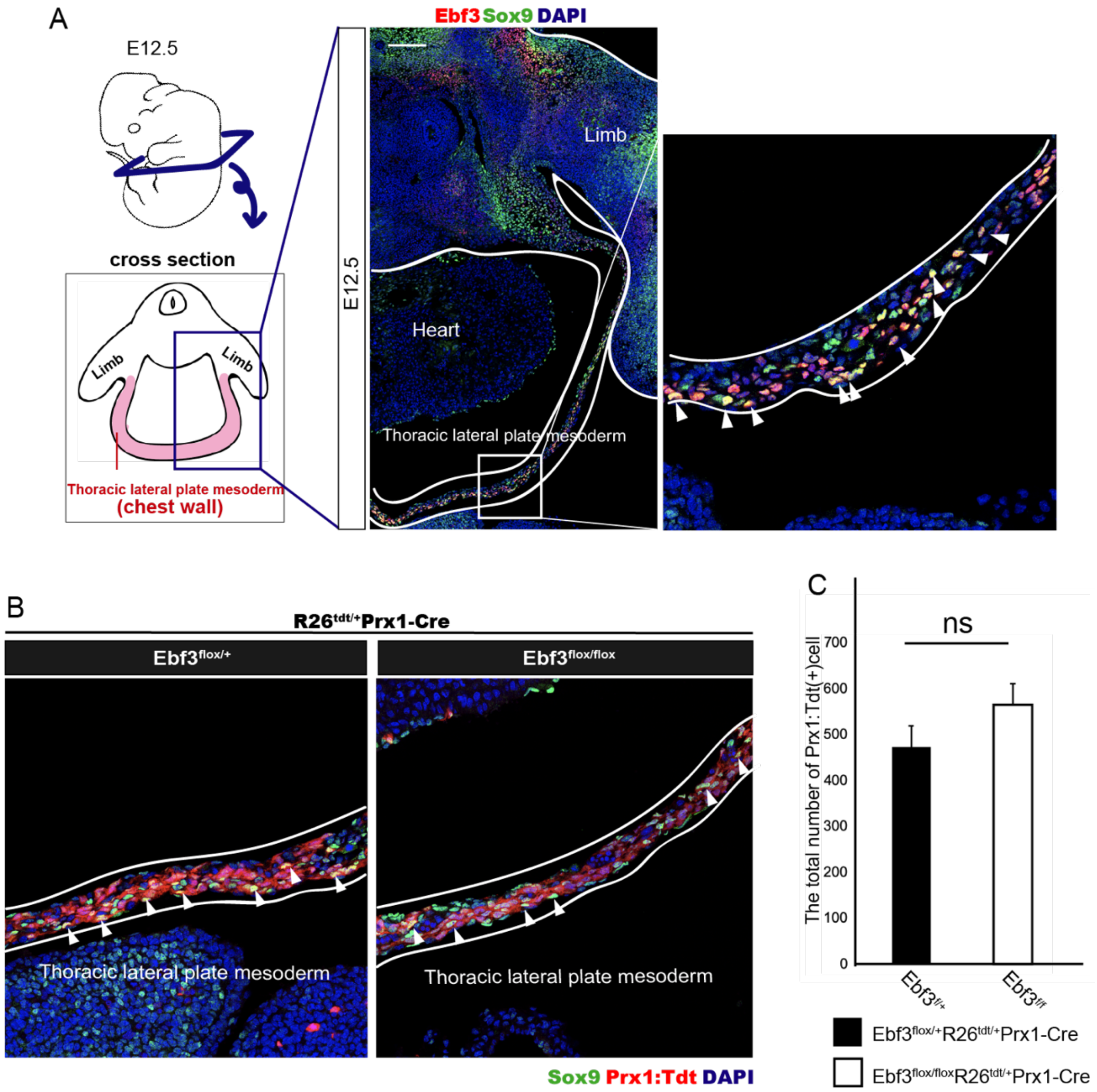

Figure S5.

The number of Sox9$^+$ cells is not changed in Ebf3-deficient thoracic lateral plate mesoderm at E12.5

A) Ebf3-expressing cells and Sox9$^+$ cells in the thoracic lateral plate mesoderm at E12.5 based on transverse sections and immunostaining with anti-Ebf3 and anti-Sox9 antibodies. White arrows show Ebf3/Sox9-double-positive cells (scale bar, 100μm).

B) Immunostaining for Sox9$^+$ cells in Prx1:Tdtomato$^+$ thoracic lateral plate mesoderm at E12.5 based on transverse sections of Ebf3$^{flox/flox}$Prx1-Cre and Ebf3$^{flox/+}$ Prx1-Cre embryos (white arrows).

C) Total number of Prx1:Tdtomato$^+$ thoracic LPMs at E12.5 based on transverse sections. Error bars represent SEM, ns: non-significant (n = 4).

Development • Supplementary information

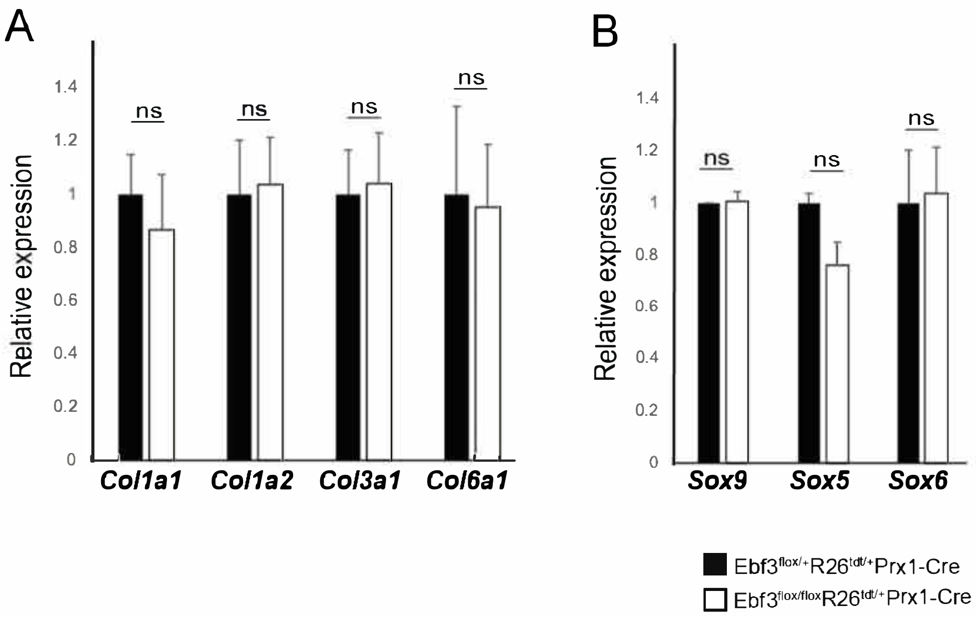

Figure S6.

Relative expression levels of collagen and Sox-family genes in Prx1-Cre Ebf3 KO mice compared to those in Prx1-Cre Ebf3 heterozygous mice

A) Relative expression levels of collagen genes in Ebf3$^{flox/flox}$Prx1-Cre embryos compared to those in Ebf3$^{flox/+}$ Prx1-Cre embryos from RNA-seq analysis, ns: non-significant.

B) Relative expression levels of Sox-family genes in Ebf3$^{flox/flox}$Prx1-Cre embryos compared to those in Ebf3$^{flox/+}$ Prx1-Cre embryos from RNA-seq analysis, ns: non-significant.

Development • Supplementary information

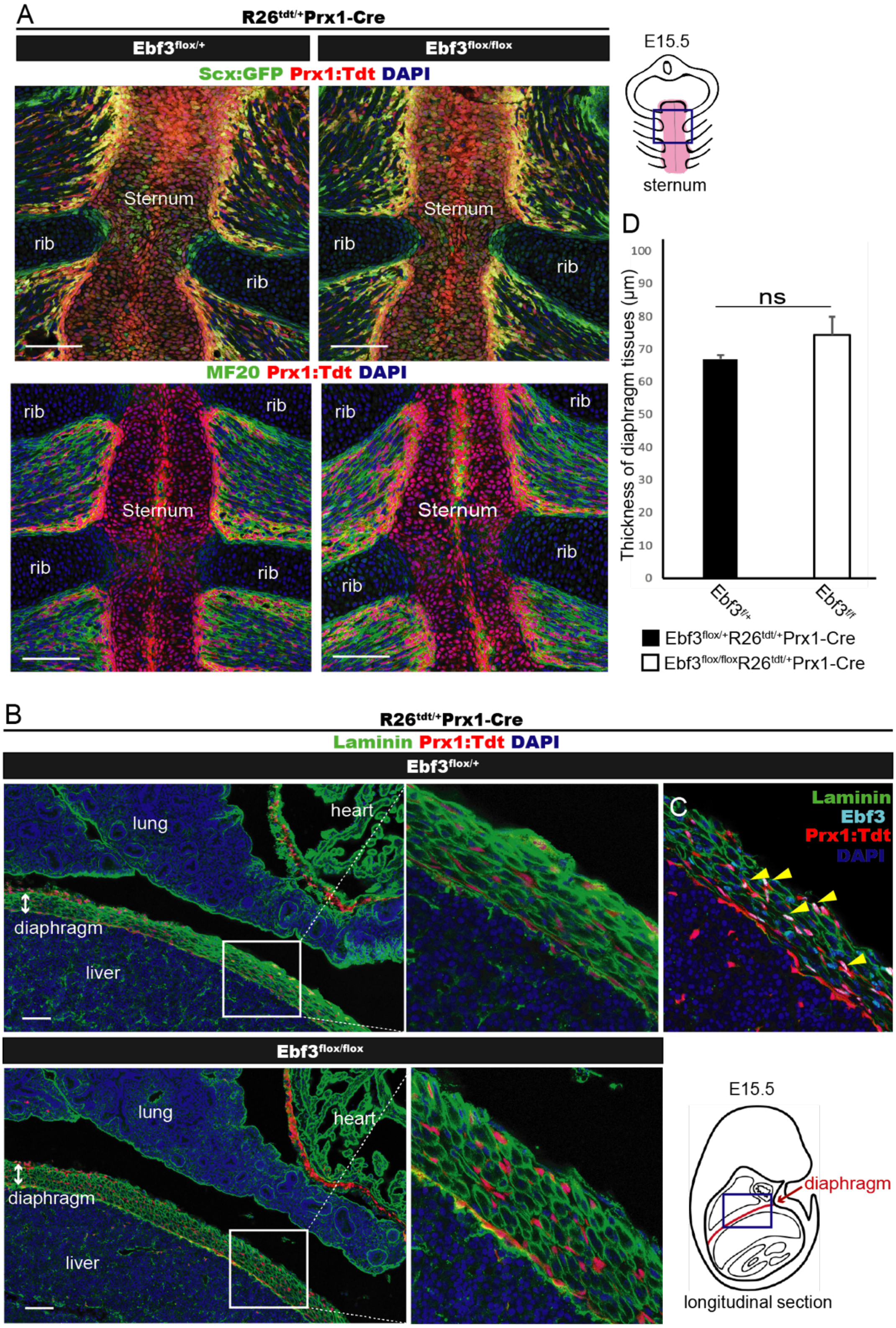
A
R26tdt/+Prx1-Cre
Ebf3flox/+
Ebf3flox/flox
Scx:GFP Prx1:Tdt DAPI
Sternum
rib
rib
MF20 Prx1:Tdt DAPI
E15.5
sternum
D
ns
Thickness of diaphragm tissues (μm)
100
90
80
70
60
50
40
30
20
10
0
Ebf3f/+
Ebf3f/f
Ebf3flox/+R26tdt/+Prx1-Cre
Ebf3flox/floxR26tdt/+Prx1-Cre
B
R26tdt/+Prx1-Cre
Laminin Prx1:Tdt DAPI
Ebf3flox/+
lung
heart
diaphragm
liver
C
Laminin
Ebf3
Prx1:Tdt
DAPI
Ebf3flox/flox
E15.5
diaphragm
longitudinal section

Figure S7.

Tendon/connective tissues and muscles surrounding the sternum and muscles in the diaphragm are normally formed in Prx1-Cre Ebf3 KO mice

A) Scx:GFP tendon-like tissues and MF20^{+} intercostal-muscles in Prx1:Tdtomato^{+} muscle connective tissues (MCT) surrounding the sternum were normally formed in Prx1-Cre Ebf3 KO mice at E15.5, on transverse section of Prx1-Cre R26$^{Tdt/+}$ (scale bar, 100μm).

B) Immunostaining for Laminin^{+} myofibers and Prx1:Tdtomato^{+} MCTs in the diaphragm at E15.5 based on longitudinal sections (scale bar, 100μm). Diaphragm tissues were similarly formed in between Ebf3$^{flox/flox}$Prx1-Cre and Ebf3$^{flox/+}$ Prx1-Cre embryos.

C) Immunostaining for Ebf3^{+} cells in Prx1:Tdtomato^{+} MCTs of the diaphragm at E15.5 based on longitudinal sections of Ebf3$^{flox/+}$ Prx1-Cre R26$^{Tdt/+}$ (yellow arrows).

D) Thickness of Laminin^{+} diaphragm tissues at E15.5 based on longitudinal sections. Error bars represent SEM, ns: non-significant (n = 3).
