# Supplementary material for: Transient and lineage-restricted requirement of Ebf3 for sternum ossification
Source: Development. 2020 May 12;147(9):dev186239. doi: 10.1242/dev.186239 (PMC7240299; doi:10.1242/dev.186239)
Supplement: Reviewer comments [file develop-147-186239_review_history.pdf]

Transient and lineage-restricted requirement of Ebf3 for sternum ossification

Mao Kuriki, Fuminori Sato, Hiroyuki N. Arai, Maina Sogabe, Mari Kaneko, Hiroshi Kiyonari, Koichi Kawakami, Yuki Yoshimoto, Chisa Shukunami and Atsuko Sehara-Fujisawa
DOI: 10.1242/dev.186239

Editor: Haruhiko Koseki

Review timeline

Original submission:	6 November 2019
Editorial decision:	30 December 2019
First revision received:	24 February 2020
Editorial decision:	19 March 2020
Second revision received:	20 March 2020
Accepted:	20 March 2020

Original submission

First decision letter

MS ID#: DEVELOP/2019/186239

MS TITLE: Transient and lineage-restricted requirement of Ebf3 for sternum ossification

AUTHORS: Mao Kuriki, Fuminori Sato, Hiroyuki Arai, Maina Sogabe, Mari Kaneko, Hiroshi Kiyonari, Koichi Kawakami, Yuki Yoshimoto, Chisa Shukunami, and Atsuko Sehara-Fujisawa

I have now received all the referees' reports on the above manuscript, and have reached a decision. The referees' comments are appended below, or you can access them online: please go to BenchPress and click on the 'Manuscripts with Decisions' queue in the Author Area.

As you will see, the referees express considerable interest in your work, but have some significant criticisms and recommend a substantial revision of your manuscript before we can consider publication. If you are able to revise the manuscript along the lines suggested, I will be happy receive a revised version of the manuscript. Particularly, reviewer 3 found it a bit difficult to follow the logical flow of the manuscript. Your revised paper will be re-reviewed by one or more of the original referees, and acceptance of your manuscript will depend on your addressing satisfactorily the reviewers' major concerns. Please also note that Development will normally permit only one round of major revision.

Please attend to all of the reviewers' comments and ensure that you clearly highlight all changes made in the revised manuscript. Please avoid using 'Tracked changes' in Word files as these are lost in PDF conversion. I should be grateful if you would also provide a point-by-point response detailing how you have dealt with the points raised by the reviewers in the 'Response to Reviewers' box. If you do not agree with any of their criticisms or suggestions please explain clearly why this is so.

© 2020. Published by The Company of Biologists under the terms of the Creative Commons Attribution License (https://creativecommons.org/licenses/by/4.0/).

Reviewer 1

Advance summary and potential significance to field

This study presents the critical function of Ebf3 for sternum ossification by a combination of multiple experimental strategies. First of all, enhancer-trap screening by zebrafish is a powerful technique to reveal a novel molecular pathway in development. Next, genetic analysis for gene deletion and lineage trance study revealed the role of Rbf3 in sternum ossification. A more detailed analysis of LPMs differentiation will be determined in a future based on this finding using tools established in this study.

Comments for the author

This is a powerful study involving enhancer screening, conditional gene deletion and lineage tracing analysis. To further confirm the molecular network in detail Ebf3 ChIP analysis is critical, however, the reviewer believes that this MS has enough solid and informative results and discussion and could be published in the present form.

Reviewer 2

Advance summary and potential significance to field

This study investigates the role of Ebf3 in sternal ossification. Starting from a screen in zebrafish, they identified as a candidate Ebf3, then moved to a conditional mouse mutant and performed a number of studies on the role of this gene in the formation of the sternum using different combinations of mutants and lineage markers. They identified a critical window during early embryogenesis when Ebf3 is required for sternal ossification and this requirement is restricted to the ossification step in development. The experiments are extensive with use of a number of mouse mutants, and well done. This study makes a strong contribution to the field of lateral plate derived sternal precursors. Some minor suggestions are made below to improve the manuscript and clarify some points.

Comments for the author

Comments :
1) Please restrict the introduction to background information and limit communicating results in this section. Ex. Remove line 42-43 and reduce details in lines 81-91 presenting the work only in general terms.
2) Fig 2B – it appears that Ebf2 is also expressed in the sternal bar as indicated in the Figure. Please clarify how this is cited in the text line 121, indicating that Ebf2 is not expressed in the lateral plate.
3) There is evidence in the literature of the role of Ebf3 in myogenesis (ex. Jin et al Nat Comm 2014; El-Magd et al Dev Growth Diff, 2013; Magli ELife 2019; Green and Vetter Dev. Biol, 2011). On line 133 the authors cite no muscle defects. They need to check carefully in the pharyngeal arches and diaphragm to see if this is indeed the case and put in the context of the literature.
4) In Fig 3B left, the ribs are stained weakly compared to Fig 2D. Please indicate if rib phenotypes in the Ebf3 full or conditional mutant are clearly lacking.
5) For the different crosses, please indicate how many controls were used (WT vs. heterozygous) and if phenotypes are clearly lacking in the Ebf3 heterozygous.
6) Line 173 – Tmx was presumably injected into pregnant mice, not embryos.
7) Fig 5D – typo "Day X"
8) Fig 6 – please indicate what criteria were used for designating the cell types following RNA-seq (how many genes, cut-of, etc.), and the confidence given that in some cases CT cells and mesenchymal cells might be confused since neither is very well defined.
9) The model in Fig 7 is not very clear on some points: a) why is JunB+ highlighted here? There is not functional data. Putting it here draws attention to this gene above other ones identified; b) 3 cell states are indicated after the progenitor. What is the evidence that these are distinct? The term myofibroblast can be misleading suggesting that it is myogenic, then gives rise to a tenocyte.

© 2020. Published by The Company of Biologists under the terms of the Creative Commons Attribution License (https://creativecommons.org/licenses/by/4.0/).

There is no evidence supporting this supposition; c) the placement of Efb3-negative is confusing – it is not clear at this stage what happens to an Ebf3+ cell.

Reviewer 3

Advance summary and potential significance to field

The MS reports a function for the transcription factor Ebf3 in sternum ossification in mice.
This MS seems to represent of lot of work, but the story was difficult to follow. I had difficulty to conciliate the endogenous expression of Ebf3 in tendon/muscle connective tissue cells, the sternum ossification defect in Ebf3 mutant mice and the Ebf3 literature, which was not always referenced. The jump from zebrafish to mouse was also not easy to follow. The sternum ossification phenotype observed in the Ebf3 mutant mice by the authors has to be related to the endogenous expression of Ebf3 and the literature.

Comments for the author

It is mentioned in the abstract that Ebf3 was identified «as a transcription factor marking tenocytes and connective tissue cells in skeletal muscle of embryos», and then «Ebf3 knockout (KO) mice had sternum ossification defects, without affecting chondrogenesis». Given the expression of Ebf3 in tenocytes and muscle connective tissue cells, the obvious question is: How are the tendons and the muscle connective tissue affected in Ebf3 knockout (KO) mice? Prx1-Cre is the perfect mouse line to follow defects in tendons and muscle connective tissues.

Why is the sternum the only bone affected in Ebf3 mutant mice? Could it be related to the diaphragm defect observed in the same mutant? The transcription factor Ebf3 as been described as a determinant of muscle cell-specific transcription in the diaphragm (Jin et al. Nat. com. 2014 PMID: 24786561). Is the sternum defect observed in this MS related to the diaphragm impairment in Ebf3 mutant mice? This work should be mentioned, referenced and discussed.

The tissue dissection for RNA-seq experiment is not clearly explained. The tissue used for RNA-seq is named thoracic lateral plate mesoderm. At E10.5 there is a limb bud, does it mean that the forelimb bud was dissected?
The basis of the classification of differentially-expressed genes in CT cells, in the 3 categories is not clear. (1) Genes required for the differentiation of connective-tissue (CT) cells, (2) Genes required for the differentiation of bone cells and osteoblasts, and (3) Genes required for the differentiation of mesenchymal cells. How was established these gene lists? The fact that there is no bone-specific gene and only 3 mesenchymal-specific genes is difficult to understand. The distinction between mesenchymal cells and connective tissue is not clear.

First revision

Author response to reviewers' comments

Response to Reviews

For Reviewer 2:

1) Please restrict the introduction to background information and limit communicating results in this section. Ex. Remove line 42-43 and reduce details in lines 81-91 presenting the work only in general terms.

Answer(A) for 1)
A-1):

© 2020. Published by The Company of Biologists under the terms of the Creative Commons Attribution License (https://creativecommons.org/licenses/by/4.0/).

In accordance with the reviewer's comment, we removed text in Introduction, line 42-43, "Here, we report an identification of a transcription factor Ebf3 essential for the sternum ossification.", and shortened text in lines 80-85 as follows: "From this idea, we screened enhancer-trap lines of zebrafish and identified the early B-cell factor3 (ebf3) as a transcription factor marking scleraxis (scx)-expressing tenocytes and MCTs of zebrafish and mouse embryos in common. Ebf3 is required for the sternum ossification. Ebf3 is required in Scx+ cell lineage of LPMs transiently during early embryonic stage. Analyses of embryonic LPMs at that stage suggest roles of EBF3 in the differentiation of osteo-fibrogenic progenitors towards Runx2+ pre-osteoblasts at the boundary of lateral and somitic mesoderms.".

2) Fig 2B – it appears that Ebf2 is also expressed in the sternal bar as indicated in the Figure. Please clarify how this is cited in the text line 121, indicating that Ebf2 is not expressed in the lateral plate.

A-2):
According to the reviewer's comment, we added the text about details of the Ebf2 expression in lines 118-119 as follows: "Ebf2 also starts to be expressed in primordial bars at E11.5 (Figure 2C).". Ebf2 is expressed in sternal bars of embryos at E11.5, but not in the thoracic lateral plate mesoderm at E10.5.

3) There is evidence in the literature of the role of Ebf3 in myogenesis (ex. Jin et al Nat Comm 2014; El-Magd et al Dev Growth Diff, 2013; Magli ELife 2019; Green and Vetter Dev. Biol, 2011). On line 133 the authors cite no muscle defects. They need to check carefully in the pharyngeal arches and diaphragm to see if this is indeed the case and put in the context of the literature.

A-3):
As we had written in the previous manuscript, no apparent defects were observed in skeletal muscle formation. In addition, because we had intended to focus on the phenotype of sternum formation, we did not discuss roles of Ebf3 in myogenesis in our previous manuscript.
However, as was pointed out by the reviewer, it would be important to carefully examine whether the absence of Ebf3 expressed in LPMs causes any defects in skeletal myogenesis even if they are subtle. We therefore investigated whether Prx1-Cre driven Ebf3 deletion affects myogenesis adjacent to LPM-derived connective tissues in detail as an additional experiment in the revised manuscript. As a result, Prx1-Cre Ebf3 KO mice exhibited substantially normal formation of intercostal muscles and the diaphragm. We added these data as Figure S7A and S7B, and added the following text in lines 151-157: "A previous study reported the defect in muscle differentiation of the diaphragm in the Ebf3 Pan-KO mouse embryo [22]. Since Ebf3 is expressed in Prx1-Cre R26RTdtomato-activated connective tissue cells of the diaphragm and intercostal muscles adjacent the sternum (Figure S4C and S7C), we examined whether the diaphragm and intercostal skeletal tissues are affected in Prx1-Cre Ebf3-KO mice. The diaphragm and intercostal muscles and tendon/connective tissues were substantially normal in Prx1-Cre Ebf3-KO embryos, implying that Ebf3 expressed in LPM-derived MCT are not involved in myogenic defects in these tissues (Figure S7A, S7B and S7D).".
We further discussed in lines 303-307 as follows: "In contrast to a previous observation of thinner diaphragm muscles in Ebf3 Pan-KO mice [22: Jin et al Nat Comm 2014], the myogenesis of diaphragm and intercostal muscle was minimally affected in Prx1-Cre LPM-specific Ebf3 KO mice, suggesting that Ebf3 expressed in somitic MCTs is required for the myogenesis or other members of the ebf family expressed redundantly in somitic and lateral plate mesoderm compensate roles of Ebf3 for myogenesis.".

4) In Fig 3B left, the ribs are stained weakly compared to Fig 2D. Please indicate if rib phenotypes in the Ebf3 full or conditional mutant are clearly lacking.

A-4):
There is no defect in rib-bone development in Ebf3 general and conditional mutants. To make the figures easy-to-grasp ones, we arranged the order of photos in Figure 2D in the same way to those in Fugure 3B: those showing the ventral side (sternum) on the left and those showing the dorsal side (ribs/vertebrae) on the right.

© 2020. Published by The Company of Biologists under the terms of the Creative Commons Attribution License (https://creativecommons.org/licenses/by/4.0/).

5) For the different crosses, please indicate how many controls were used (WT vs. heterozygous) and if phenotypes are clearly lacking in the Ebf3 heterozygous.

A-5):
The sternum ossification was not affected in all the Ebf3 heterozygous mice. In accordance with reviewer's comment, we described numbers of samples examined clearly.
in lines 128-132 as follow: "Alizarin red and Alcian blue staining revealed that Ebf3-KO embryos had defects in sternal-bone formation, while sternal chondrogenesis occurs normally in these mice, with the exception of deformations in the first segment of the sternum (5/5 embryos) (Figure 2E). Sternal-ossification and chondrogenesis defects were not observed in CAG-Cre Ebf3 heterozygous mice (4/4 embryos).",
in lines 139-142 as follow: "Prx1-Cre Ebf3-KO embryos showed defects in sternum ossification (6/6 embryos), similar to that observed in CAG-Cre general Ebf3-KO embryos (Figure 3B), while no defects were observed in Prx1-Cre Ebf3 heterozygous mice (10/10 embryos), indicating that the Ebf3 expressed in LPMs is required for sternum ossification.",
in line 182-184 as follow: "Sternum phenotypes were reproducible only when tamoxifen was injected into these mice at E9.5 (5/5 embryos) or E10.5 (4/5 embryos), but not at E8.5 (6/7 embryos) E11.5 (3/3 embryos), E12.5 (7/7 embryos) or in control (Ubc-CreERT2 Ebf3 heterozygous and wild-type) mice,",
in lines 237-239 as follow: "To determine whether Ebf3 is required in Scx+ LPMs, Ebf3flox/flox mice were crossed with Ebf3flox/+ Scx-Cre mice and found defective sternum ossification in Ebf3flox/flox Scx-Cre mice (6/6 embryos) but not in the heterozygous mice (7/7 embryos).".

6) Line 173 – Tmx was presumably injected into pregnant mice, not embryos.

7) Fig 5D – typo "Day X"

A-6) and 7):
Thanks to the reviewer's comment, we clearly indicated that Tmx was injected into "pregnant dams" in the line 180 and the line 361, and fixed typos "day X" and "day Y" into "Day X" and "Day Y" in Figure 5B.

8) Fig 6 – please indicate what criteria were used for designating the cell types following RNA-seq (how many genes, cut-of, etc.), and the confidence given that in some cases CT cells and mesenchymal cells might be confused since neither is very well defined.

A-8):
Thanks to the reviewer's comment, we described the way of analyses more clearly.
We carried out the cell-type categorization by annotation methods of IPA software analysis. To make this process clearer, we added the following text to Result in lines 194-201 as follow: "By using IPA software (Ingenuity Pathway Analysis), downstream analysis was performed for this RNA-seq data. In the IPA Knowledge Base derived from omics data, annotated genes were categorized by their biological functions. In the category of "Cellular Development", we focused on three groups associated with the differentiation of (1) connective tissue (CT) cells, (2) bone cells/osteoblasts, and (3) mesenchymal cells (Figure 6B). The Venn diagram in Figure 6C shows the differentially expressed genes annotated in these three groups, in which Red and Blue-colored genes were those significantly up- and down-regulated, respectively, in Prx1-Cre Ebf3 KO mice (Figure 6C).".
We also added information about criteria used in IPA analysis (Cut Off, P-value) and the number of molecules picked upped in each cell-type group in Figure 6B.

9) The model in Fig 7 is not very clear on some points: a) why is JunB+ highlighted here? There is not functional data. Putting it here draws attention to this gene above other ones identified; b) 3 cell states are indicated after the progenitor. What is the evidence that these are distinct? The term myofibroblast can be misleading suggesting that it is myogenic, then gives rise to a tenocyte. There is no evidence supporting this supposition; c) the placement of Efb3-negative is confusing – it is not clear at this stage what happens to an Ebf3+ cell.

A-9):
We thank the reviewer for this comment. It helped us significantly improve the model drawn in Figure 7C. In short, we simplified the model in Figure 7C based on the data we obtained in this

© 2020. Published by The Company of Biologists under the terms of the Creative Commons Attribution License (https://creativecommons.org/licenses/by/4.0/).

study. Gene names of JunB and EGFR, regulators for the osteoblast differentiation (Kenner L et al Cell Biol 2004, Sibilia M et al Development 2003), of which expression changed significantly in Ebf3 KO mice, were removed from this diagram to avoid the complexity. The term “fibroblastic cells” was used instead of myofibroblasts. We further discussed in lines 311-317 as follows: “The number of Islet1+ mesenchymal cells and expressions of connective tissue cell/tenocyte-associated genes such as Egr1, Egr2, and Osr1were increased in Ebf3-deleted thoracic LPMs. How Ebf3 regulates production of Islet1+ cells and the fate decision of LPMs into osteoblasts and fibroblasts remain open questions. Identifications of target genes for Ebf3, for example by CHIP analyses, together with single cell analyses of LPMs at the early embryonic stages of wild type and Ebf3 KO mice are essential for more detailed understanding the function of Ebf3 in the LPMs differentiation.”.

For Reviewer 3:

Advance summary and potential significance to field
The MS reports a function for the transcription factor Ebf3 in sternum ossification in mice. This MS seems to represent of lot of work, but the story was difficult to follow. I had difficulty to conciliate the endogenous expression of Ebf3 in tendon/muscle connective tissue cells, the sternum ossification defect in Ebf3 mutant mice and the Ebf3 literature, which was not always referenced. The jump from zebrafish to mouse was also not easy to follow. The sternum ossification phenotype observed in the Ebf3 mutant mice by the authors has to be related to the endogenous expression of Ebf3 and the literature.

For this comment:
The screening of zebrafish enhancer-trap lines led us to successfully identify Ebf3 as a novel gene marker in embryonic tenocytes and connective-tissue cells and find its roles in differentiation of lateral plate mesenchymal cells into the sternal bone in mice. We therefore believe that the zebrafish is the valuable vertebrate model to investigate development of the muscle-skeletal system.
However, we read Reviewer 3’s profound comments very carefully and realized that it is essential to add the data on the phenotypes of skeletal muscles and tendon/connective tissues referring the literature and to show the data on expression of Ebf3 more clearly using Prx1-Cre R26RTdtomato transgenic mice to indicate relationship between expression patterns of Ebf3 and phenotypes of its KO mice.

1) On the phenotypes in skeletal muscles and tendon/connective tissues
According to the reviewer 3’s advice, we examined whether skeletal muscle and tendon/connective tissues defects are observed in Prx1-Cre Ebf3 KO mice in the revised version of manuscript in order to know whether Ebf3 plays a role in the formation of tendon tissues where Ebf3 is expressed and the formation of skeletal muscle that attach to those tendons. As a result, both formation of rib muscles and the tendons connecting ribs to the sternum and formation of the diaphragm muscle were not affected significantly. The data were shown in Figure S7A, and described in text, in which we also mentioned the previous work, as follows (in lines 151-157): “A previous study reported the defect in muscle differentiation of the diaphragm in the Ebf3 Pan-KO mouse embryo [22]. Since Ebf3 is expressed in Prx1-Cre R26RTdtomato-activated connective tissue cells of the diaphragm and intercostal muscles adjacent the sternum (Figure S4C and S7C), we examined whether the diaphragm and intercostal skeletal tissues are affected in Prx1-Cre Ebf3-KO mice. The diaphragm and intercostal muscles and tendon/connective tissues were substantially normal in Prx1-Cre Ebf3-KO embryos, implying that Ebf3 expressed in LPM-derived MCT are not involved in myogenic defects in these tissues (Figure S7A, S7B and S7D).”.

2)On the expression of Ebf3 in embryonic tendons and connective tissues
In order to show that Ebf3 is expressed in non-muscle tissues including the tendon and non-myogenic mesenchymal cells adjacent to myotomes at the early embryonic stage in mice, similarly to the expression patterns of Ebf3 in zebrafish embryos, the expression pattern of Ebf3 in the transverse sections of Prx1-Cre R26RTdtomato mice was indicated in the new version of manuscript.
As shown in Figure 2B, somitic Ebf3 is expressed in syndetomes adjacent to myotomes stained with anti-Pax7 Ab, while Prx1-Cre Tdt+ lateral plate mesenchymes found ventrally to myotomes also express Ebf3 at E10.5, the stage prior to the migration of Pax7-positive myogenic cells into the lateral plate mesoderm. Although mouse tissues are much more complicated than those of

© 2020. Published by The Company of Biologists under the terms of the Creative Commons Attribution License (https://creativecommons.org/licenses/by/4.0/).

zebrafish, the result indicates that mouse and zebrafish embryos largely share spatial expression patterns of Ebf3: clusters of tenocytes and connective tissue cells adjacent to myotomes. We hope that these data can help readers jump from zebrafish to mice more easily in this manuscript than in the previous one.
We also showed expression of Ebf3 in Prx1-Cre R26RTdtomato-positive cells in the tendon/connective tissues surrounding the sternum and in the diaphragm muscle later at E18.5 in Figure S4C and Figure S7C, respectively. We showed these data together with description in lines 161-163 as follows: “Ebf3 is expressed in the periosteum and Prx1-Cre R26RTdtomato-activated tendon/connective tissue cells surrounding the sternum, and connective tissue cells interspersed among intercostal myofibers at the ossification stage (E18.5) (Figure S4A and S4C).” and in lines 152-153 as follows: “Since Ebf3 is expressed in Prx1-Cre R26RTdtomato-activated connective tissue cells of the diaphragm and intercostal muscles adjacent the sternum (Figure S4C and S7C),”, respectively.

Comment 1:
How are the tendons and the muscle connective tissue affected in Ebf3 knockout (KO) mice? Prx1-Cre is the perfect mouse line to follow defects in tendons and muscle connective tissues.

Answer1(A1):
As was mentioned above, we added data Figure S7A and S7B in the revised manuscript, showing that both the tendon and muscle connective tissue were not affected in Prx1-Cre R26RTdtomato mice.

Comment 2:
Why is the sternum the only bone affected in Ebf3 mutant mice? Could it be related to the diaphragm defect observed in the same mutant? The transcription factor Ebf3 as been described as a determinant of muscle cell-specific transcription in the diaphragm (Jin et al. Nat. com. 2014 PMID: 24786561). Is the sternum defect observed in this MS related to the diaphragm impairment in Ebf3 mutant mice? This work should be mentioned, referenced and discussed.

A2:
We are also interested in the result that ossification defects in Ebf3 KO mice were specific to the sternum and metatarsal bones. As shown with in situ hybridization, other members of Ebf family are also expressed in the somite and limb buds. We therefore think that those genes complement deletion of Ebf3 in the somite and limb buds.
We further added new data on the expression patterns of Ebf3 at E10.5 and E18.5 in addition to E12.5 in the new version of our manuscript (Figure 2B and Figure S4C). These data indicate that Ebf3 is expressed in non-muscle cells adjacent to muscle fibers throughout the embryogenesis. We did not refer to the previously report, Jin et al. Nat. com. 2014 PMID 24786561, because we were not brave enough to point out that their data were too rough and careless. However, thanks to the reviewer’s advice, we shortly referred to it in the revised version of manuscript.
Based on these results, discussion was added in the new version of manuscript in lines 300-307 as follows: “In this study, we found critical roles of Ebf3 in the sternum ossification. Whereas members of the ebf family are expressed redundantly in the somitic mesoderm and limb buds, expression of Ebf3 dominates the others in thoracic LPMs, which should lead to the defective sternum ossification by the lack of Ebf3 in LPMs. In contrast to a previous observation of thinner diaphragm muscles in Ebf3 Pan-KO mice [22: Jin et al Nat Comm 2014], the myogenesis of diaphragm and intercostal muscle was minimally affected in Prx1-Cre LPM-specific Ebf3 KO mice, suggesting that Ebf3 expressed in somitic MCTs is required for the myogenesis or other members of the ebf family expressed redundantly in somitic and lateral plate mesoderm compensate roles of Ebf3 for myogenesis.”.

Comment 3:
The tissue dissection for RNA-seq experiment is not clearly explained. The tissue used for RNA-seq is named thoracic lateral plate mesoderm. At E10.5 there is a limb bud, does it mean that the forelimb bud was dissected?

A3:
We added the following text to Result (in lines 190-192): “Trunks without limb buds, heart and other organs in thoracic /abdominal cavity were isolated, and then thoracic wall tissues were

© 2020. Published by The Company of Biologists under the terms of the Creative Commons Attribution License (https://creativecommons.org/licenses/by/4.0/).

excised. Prx1-Cre R26RTdtomato-activated LPMs were isolated from these thoracic wall tissues by fluorescence-activated cell sorting (FACS).”.

Comment 4:
The basis of the classification of differentially-expressed genes in CT cells, in the 3 categories is not clear. (1) Genes required for the differentiation of connective-tissue (CT) cells, (2) Genes required for the differentiation of bone cells and osteoblasts, and (3) Genes required for the differentiation of mesenchymal cells. How was established these gene lists? The fact that there is no bone-specific gene and only 3 mesenchymal-specific genes is difficult to understand. The distinction between mesenchymal cells and connective tissue is not clear.

A4:
We carried out the cell-type categorization by gene annotation methods of IPA software analysis. To make the process of categorization clearer, we added the following text to Result (in lines 194-201): “By using IPA software (Ingenuity Pathway Analysis), downstream analysis was performed for this RNA-seq data. In the IPA Knowledge Base derived from omics data, annotated genes were categorized by their biological functions. In the category of “Cellular Development”, we focused on three groups associated with the differentiation of (1) connective tissue (CT) cells, (2) bone cells/osteoblasts, and (3) mesenchymal cells (Figure 6B). The Venn diagram in Figure 6C shows the differentially expressed genes annotated in these three groups, in which Red and Blue-colored genes were those significantly up- and down-regulated, respectively, in Prx1-Cre Ebf3 KO mice (Figure 6C).”. We also added information about criteria used in IPA analysis (Cut Off, P-value) and the number of molecules picked up in each cell-type group in Figure 6B.

Second decision letter

MS ID#: DEVELOP/2019/186239

MS TITLE: Transient and lineage-restricted requirement of Ebf3 for sternum ossification

AUTHORS: Mao Kuriki, Fuminori Sato, Hiroyuki Arai, Maina Sogabe, Mari Kaneko, Hiroshi Kiyonari, Koichi Kawakami, Yuki Yoshimoto, Chisa Shukunami, and Atsuko Sehara-Fujisawa

I have now received all the referees reports on the above manuscript, and have reached a decision. The referees' comments are appended below, or you can access them online: please go to BenchPress and click on the 'Manuscripts with Decisions' queue in the Author Area.

The overall evaluation is positive and we would like to publish a revised manuscript in Development, provided that the referees' comments can be satisfactorily addressed. Please attend to all of the reviewers' comments in your revised manuscript and detail them in your point-by-point response. If you do not agree with any of their criticisms or suggestions explain clearly why this is so.

Reviewer 1

Advance summary and potential significance to field

Identification of Ebf3 as a transcription factor marking tenocytes and connective tissue cells in skeletal muscle of embryos should provide us a basis for developmental biology.

Comments for the author

The authors sufficiently addressed my comments and modified the manuscript accordingly.

© 2020. Published by The Company of Biologists under the terms of the Creative Commons Attribution License (https://creativecommons.org/licenses/by/4.0/).

Reviewer 2

Advance summary and potential significance to field

The authors identify Ebf3 expressed in lateral plate mesenchyme cells, specifically in osteo-fibrogenic progenitors, as a critical regulator of sternum ossification.

Comments for the author

The authors have revised the manuscript according to comments made and I have not further major comments.

Minor points:
Please check the last sentence of the abstract which is too long and reads a bit confused.

- Also gene nomenclature needs homogenisation (Ebf3 vs. ebf3 in text; Cre vs. cre in Fig S7). Please check whole document and unify nomenclature.
- Note that Pax7 is not a conventional myotome marker, it marks the dermomyotome and not all the cells in the myotome. MyoD/Desmin are better markers to reveal the whole myotome. Please correct text accordingly.
- The newly added Jin et al. reference is incomplete.

Reviewer 3

Advance summary and potential significance to field

The MS reports a function for the transcription factor Ebf3 in sternum ossification in mice.

Comments for the author

The revised MS is easier to follow and the diagram Figure 7C is also easier to understand.

A confusion exists throughout the MS in the terminology. Tendon and connective tissue are not separate entities, whereas the systematic presentation of descriptions of “tendon” on the one hand and “connective tissue” on the other suggests, erroneously, that they are. The authors should carefully specify which connective tissue they are describing in contrast with tendons.

And I still have similar interrogation as for the previous MS:
The explanation why Prx1-Cre Ebf3flox/flox mice do not have a diaphragm muscle phenotype versus the published Ebf3KO mice is not so clear to me. Authors give an explanation for this discrepancy about redundancy “other members of the ebf family expressed redundantly in somitic and lateral plate mesoderm compensate roles of Ebf3 for myogenesis ». But redundancy should also works for Ebf3KO mice and not only for Prx1-Cre Ebf3flox/flox. Does CAG-Cre Ebf3 flox/flox mice have a muscle phenotype like Ebf3KO mice?

I still do not understand how was established the gene lists in the 3 Inginuity pathways
(1) Genes required for the differentiation of connective tissue (CT) cells,
(2) Genes required for the differentiation of bone cells and osteoblasts and (3) Genes required for the differentiation of mesenchymal cells.
I would love to have a list of genes required for CT differentiation. The fact that there is no bone-specific gene in the bone list is difficult to understand. The distinction between mesenchymal cells and connective tissue is not clear either.

I could not find the methods for qPCR (Figure 6D) nor the statistics.

In the response to comment 1:
As was mentioned above, we added data Figure S7A and S7B in the revised manuscript, showing that both the tendon and muscle connective tissue were not affected in Prx1-Cre R26RTdtomato mice.

© 2020. Published by The Company of Biologists under the terms of the Creative Commons Attribution License (https://creativecommons.org/licenses/by/4.0/).

I supposed the author meant Prx1-Cre Eff3flox/flox mice.

Page 6 line 206
osmic stress resistant (Osr1)
This is the wrong name for the main marker of muscle connective tissue. It should be Odd skipped related gene 1!

The same Spelling mistakes remains in Figure 6 in the word diffrerntiation

In Figure 7 the word expression after Ebf3 is strange

Second revision

Author response to reviewers' comments

For Reviewer 2:
Minor points:
Please check the last sentence of the abstract which is too long and reads a bit confused.

1) Also gene nomenclature needs homogenisation (Ebf3 vs. ebf3 in text; Cre vs. cre in Fig S7). Please check whole document and unify nomenclature
Answer-1) Although we used words “ebf3” and “Ebf3” to distinguish the gene and protein, respectively in previous manuscript, such expression could be misleading. We therefore unified the nomenclatures into “Ebf3” and “Cre”.

2) Note that Pax7 is not a conventional myotome marker, it marks the dermomyotome and not all the cells in the myotome. MyoD/Desmin are better markers to reveal the whole myotome. Please correct text accordingly.
A-2) According to the reviewer’s comment, we changed the words “Pax7+ myotomes” to “Pax7+ dermomyotomes” in lines 111and 115 of Result and lines 609 and 611 of Figure 2 legend.

3) The newly added Jin et al. reference is incomplete.
A-3) In accordance with the reviewer’s comment, we corrected reference of the newly added paper by Jin et al to perfect formatting in lines 516-518.

For Reviewer 3:

1) A confusion exists throughout the MS in the terminology. Tendon and connective tissue are not separate entities, whereas the systematic presentation of descriptions of “tendon” on the one hand and “connective tissue” on the other suggests, erroneously, that they are. The authors should carefully specify which connective tissue they are describing in contrast with tendons.
A-1) According to the reviewer’s comment, we defined the connective tissues at the boundary between bone and muscle as “tendon”, and those interspersed in skeletal muscles as “muscle connective tissue (MCT)” in the revised manuscript. The term, MCT, is quite popular recently.

2)And I still have similar interrogation as for the previous MS:
The explanation why Prx1-Cre Ebf3flox/flox mice do not have a diaphragm muscle phenotype versus the published Ebf3KO mice is not so clear to me. Authors give an explanation for this discrepancy about redundancy “other members of the ebf family expressed redundantly in somitic and lateral plate mesoderm compensate roles of Ebf3 for myogenesis ». But redundancy should also work for Ebf3KO mice and not only for Prx1-Cre Ebf3flox/flox. Does CAG-Cre Ebf3 flox/flox mice have a muscle phenotype like Ebf3KO mice?
A-2) We didn’t find obvious phenotypes of the muscle formation in CAG-Cre Ebf3 KO mice.
The paper by Jin et al used null Ebf3 mutated mice, which could be the reason of different muscle phenotypes between null Ebf3 mutant and CAG-Cre Ebf3 KO mice. We minimized the discussion on this discrepancy in order to focus our study on the sternum formation.

© 2020. Published by The Company of Biologists under the terms of the Creative Commons Attribution License (https://creativecommons.org/licenses/by/4.0/).

3) I still do not understand how was established the gene lists in the 3 Inginuity pathways
(1) Genes required for the differentiation of connective tissue (CT) cells,
(2) Genes required for the differentiation of bone cells and osteoblasts,
and (3) Genes required for the differentiation of mesenchymal cells.
I would love to have a list of genes required for CT differentiation. The fact that there is no bone-specific gene in the bone list is difficult to understand. The distinction between mesenchymal cells and connective tissue is not clear either.
A-3) In IPA software (Qiagen), these gene lists are constructed from GOs that belongs to "Biological Process". The list of genes categorized in GOs of Differentiation of CT cell, bone cell/fibroblast and mesenchymal cells can be extracted from GOs shown below.
Genes categorized in "Differentiation of connective tissue cells (GO) "
ID: GO:0061448
https://www.ebi.ac.uk/QuickGO/term/GO:0061448
Genes categorized in "Differentiation of bone cells (GO)"
ID: GO:0098751
https://www.ebi.ac.uk/QuickGO/term/GO:0098751
Genes categorized in "Differentiation of osteoblasts (GO)"
ID: GO:0001649, GO:0045667
https://www.ebi.ac.uk/QuickGO/term/GO:0001649
https://www.ebi.ac.uk/QuickGO/term/GO:0045667
Genes categorized in "Differentiation of mesenchymal cells (GO)"
ID: GO:0048762
https://www.ebi.ac.uk/QuickGO/term/GO:0048762

4)I could not find the methods for qPCR (Figure 6D) nor the statistics.
A-4) The data in Figure 6D is derived from RNA-seq analysis. Thus, qPCR was not performed. For easier understanding, we added the text "(from RNA-seq data)" in legend of Figure 6.

5) Page 6 line 206
osmic stress resistant (Osr1)
This is the wrong name for the main marker of muscle connective tissue. It should be Odd skipped related gene 1!
A-5) Thank you for pointing out our mistake. The full name of Osr1 was corrcted as "Odd skipped related gene 1 (Osr1)" in lines 206-207.

6) The same Spelling mistakes remains in Figure 6 in the word diffrerntiation
In Figure 7 the word expression after Ebf3 is strange
A-6) The spelling in Figure 6 were corrected and the misleading word "expression" in Figure 7C was removed.

Third decision letter

MS ID#: DEVELOP/2019/186239

MS TITLE: Transient and lineage-restricted requirement of Ebf3 for sternum ossification

AUTHORS: Mao Kuriki, Fuminori Sato, Hiroyuki N Arai, Maina Sogabe, Mari Kaneko, Hiroshi Kiyonari, Koichi Kawakami, Yuki Yoshimoto, Chisa Shukunami, and Atsuko Sehara-Fujisawa
ARTICLE TYPE: Research Article

I am happy to tell you that your manuscript has been accepted for publication in Development, pending our standard ethics checks.

© 2020. Published by The Company of Biologists under the terms of the Creative Commons Attribution License (https://creativecommons.org/licenses/by/4.0/).
